# TRIM21 modulates stability of pro-survival non-coding RNA vtRNA1–1 in human hepatocellular carcinoma cells

**DOI:** 10.1371/journal.pgen.1011614

**Published:** 2025-03-17

**Authors:** EunBin Kong, Norbert Polacek

**Affiliations:** Department for Chemistry, Biochemistry and Pharmaceutical Sciences, University of Bern, Bern, Switzerland; Huazhong University of Science and Technology Tongji Medical College, CHINA

## Abstract

Recent studies expanded our knowledge of diverse pro-survival functions of short non-coding vault RNAs. One of the human vault RNA paralogs, vtRNA1-1, modulates several intracellular processes, including proliferation, apoptosis, autophagy, and drug resistance in various types of human cancer cells. However, protein interaction partners and mechanisms by which vtRNA1-1 levels are controlled within the cells remained elusive. Here, we describe a regulatory process for vtRNA1-1 stabilization mediated by the newly identified interacting proteins, TRIM21 and TRIM25, in human hepatocellular carcinoma (HCC) cells. Depleting TRIM21 or TRIM25 reduced the stability of vtRNA1-1 both *in vivo* and *in vitro*. We also identified the responsible sequence of vtRNA1-1 for the stability regulation by TRIM21 and TRIM25 and revealed another critical factor for vtRNA1-1 stability, an NSUN2-mediated methylation at C69 of vtRNA1-1. Consequently, our findings demonstrated that the TRIM proteins govern the stability of vtRNA1-1 depending on its methylation status in HCC cells. Since vtRNA1-1 is crucial for pro-survival characteristics in HCC cells, insight into vtRNA1-1 protein binding partners and the regulation of its stability can impact the development of new anticancer strategies.

## Author summary

The significance of short non-coding RNA (ncRNA) across various fields of biology has been well established. Among these, vtRNA1-1, one of the four human vault RNA paralogs, is a short but intriguing ncRNA possessing multifaceted roles in regulating cell survival. In this study, we illuminate a novel regulatory mechanism governing vtRNA1-1 stability in human hepatocellular carcinoma (HCC) cells. By analyzing vtRNA-protein complexes, we identify two E3-ligase proteins, namely TRIM21 and TRIM25, which interact with vtRNA1-1 and promote its stability. Additionally, we identified the critical nucleotide sequence required for its stabilization and uncover the role of NSUN2-mediated modification in stabilizing vtRNA1-1. In view of the fact that vtRNA1-1 and these two TRIM proteins are upregulated in liver cancer tissues, it is reasonable to assume that this RNA-protein complex makes an important contribution to liver cancer progression. Furthermore, we demonstrate that depleting TRIM21 or TRIM25 levels in HCC cells enhances sorafenib cytotoxicity, highlighting this vtRNA-protein complex as valuable target for a potential future anticancer strategy.

## Introduction

Recent studies of non-coding RNAs (ncRNAs) have shed new light on the flow of genetic information, even as the central dogma of molecular biology is still widely accepted. Across all three domains of life, a plethora of ncRNAs, transcripts that are not translated into proteins, are expressed from genomes to perform a variety of crucial cellular functions. Over the past years, regulatory functions of diverse ncRNAs, including long ncRNA, miRNA, snoRNA, siRNA, piRNA, and tRNA-derived RNA (tDR), have been identified. These various ncRNA ribo-regulators have in common the ability to fine-tune gene expression at all levels and hence have been implicated in disease in case of misregulation [1,2].

Vault RNA (vtRNA), a distinct class of short ncRNA found in many eukaryotes, was initially considered as central component of the vault complex, a huge, barrel-shaped ribonucleoprotein (RNP) [3]. Subsequent work changed this perception since it was demonstrated that most of the vtRNA transcripts are not associated with the vault complex, thus hinting at vault complex-independent roles within cells [4,5]. It has been reported that the number and characteristics of vtRNAs vary among species [6–8]. In human cells, for instance, four paralogs of vtRNAs (vtRNA1-1, vtRNA1-2, vtRNA1-3, and vtRNA2-1), each approximately 100 nucleotides (nt) in length, are transcribed by RNA polymerase III with type II promoters, whereas mouse cells express only a single mvtRNA of 143 nt in length. Moreover, vtRNAs are missing in several widely used model organisms such as *S. cerevisiae, C. elegans*, and *D. melanogaster* [9].

To date, vtRNA1-1, which is 98 nt in length, is the most extensively studied among the three human vtRNA1 paralogs. In several human cell lines, including HeLa, HEK293, BL41, and BL2, vtRNA1-1 is considered a pro-survival factor that inhibits apoptosis and accelerates cell proliferation [10,11]. Deleting vtRNAs leads to the misregulation of MAPK signaling, a renowned pro-survival pathway regulating key cellular mechanisms for cell survival. In addition, vtRNA1-1 regulates autophagy, an important process for maintaining cellular homeostasis, in two different ways. In hepatocellular carcinoma (HCC) cell line Huh7, vtRNA1-1 is considered a positive regulator of the global autophagic flux by modulating lysosomal characteristics, such as maintaining an acidic pH, through transcriptional regulation of lysosomal genes. vtRNA1-1 inhibits the ERK-TFEB signaling axis, thereby orchestrating the expression of coordinated lysosomal expression and regulation (CLEAR) network genes involved in lysosome biogenesis and function [12]. On the other hand, it has been proposed that autophagy mediated by p62/SQSTM, a well-known receptor for selective autophagy, is negatively regulated by this ncRNA through direct interaction with vtRNA1-1. It has been suggested that vtRNA1-1 inhibits multimerization of p62/SQSTM, a key step of p62-dependent autophagy, thereby impairing the autophagic degradation of cargo [13]. Since autophagy plays a pivotal role in drug resistance in anticancer therapy [14], vtRNA1-1 is also implicated in the cytotoxicity of the antitumor drugs in HCC cells. Indeed, we demonstrated recently that loss of vtRNA1-1 expression in HCC cells potentiates the cytotoxicity of Sorafenib *in vivo* and *in vitro* [12]. Furthermore, vtRNA1-1 regulates gene expression involved in the epidermal differentiation program. NSUN2 (NOP2/Sun RNA methyltransferase 2) mediates methylation of vtRNA1-1 at cytosine 69 and the methylation status of vtRNA1-1 determines production of vtRNA1-1-derived small RNA (svRNA). These svRNAs are associated with the regulation of cellular differentiation through a miRNA-like function [15,16].

Here, we disclose a novel process regulating vtRNA1-1 stability in human HCC cells. Although the significance of vtRNA1-1 in cell viability and tumorigenicity has already been established [7,10–12], the mechanism that controls vtRNA1-1 levels within the cells remains unknown. Therefore, investigating how cellular vtRNA1-1 levels are regulated is a valuable topic that offers deeper insights into its biology. Given that the expression of vtRNA is markedly upregulated in tumor tissues derived from liver cancer patients compared to surrounding healthy tissues [7], comprehensive understanding of the mechanisms governing vtRNA1-1 abundance would also be beneficial to cancer biology. We uncovered two innate immunity-related E3 ligase proteins, TRIM21 and TRIM25, of interacting with vtRNA1-1 and regulating its cellular stability. Depleting TRIM21 or TRIM25 in HCC cells reduces vtRNA1-1 stability both *in vivo* and *in vitro*. Additionally, we identified the specific nucleotides in vtRNA1-1 responsible for the stability and clarified the relationship between m^5^C69 modification of vtRNA1-1 mediated by NSUN2 and its stability. Collectively, these findings unveil a novel regulatory mechanism for vtRNA1-1 stability, which can have notable implications in tumorigenesis and drug resistance, thus presenting a potential strategy for anti-cancer therapy in hepatocellular carcinoma.

## Results

### Vault RNA1-1 interacts with TRIM21 and TRIM25

**Fig 1:**
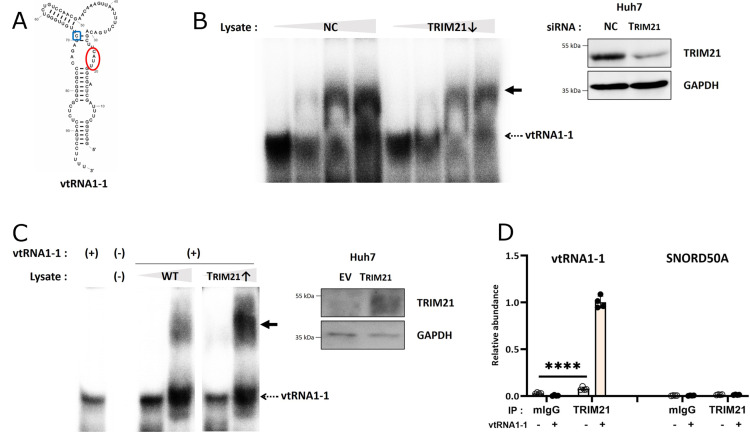
Vault RNA1-1 interacts with TRIM21. (A) A scheme of predicted vtRNA1-1 secondary structure. C69 (methylated by NSUN-2) and residues 21-24 (mutated in the M10 variant) are highlighted in blue and red, respectively. (B) EMSA of radiolabeled vtRNA1-1 with increasing concentration of cell lysate obtained from Huh7 vtRNA1-1 knock-out (KO) cells that were transfected with siRNA targeting TRIM21 or that were mock transfected (NC) (left). The bold arrow marks the upshifted RNP. Immunoblot analysis using the indicated antibodies confirmed the TRIM21 knockdown in the lysate used for the EMSA (right). NC denotes the negative control (mock transfection). GADPH served as a loading control. (C) EMSA of radiolabeled vtRNA1-1 with increasing concentration of cell lysate obtained from Huh7 vtRNA1-1 KO cells overexpressing TRIM21 (left). Immunoblot analysis using the indicated antibodies confirmed TRIM21 overexpression of lysate for the EMSA (right). GAPDH served as a loading control and EV denotes the empty vector. (D) fCLIP using the indicated antibodies (mIgG, TRIM21) and lysates obtained from cells either expressing vtRNA1-1 (+) or not (-). Co-immunoprecipitation was assessed by RT-qPCR using primers specific for vtRNA1-1 or SNORD50A. Error bars indicate the standard deviation. ****p < 0.0001 (two-way ANOVA test).

The function of ncRNAs in cells is determined by their interactions with other nucleic acids or proteins. Numerous ncRNAs interact with unique binding partners to perform specific roles. Over the past decades, the importance of RNA-protein interactions in modulating various cellular processes has become increasingly clear. As a potent regulator of cell viability and tumorigenicity, vtRNA1-1 (Fig 1A) interact with several proteins, such as p62/SQSTM and SRSF2 [13,16]. We confirmed the interaction between vtRNA1-1 and proteins in human hepatocellular carcinoma cell line Huh7 using RNA EMSA. Increasing the concentration of cell lysate resulted in an upshift of 5’-radiolabeled *in vitro*-transcribed vtRNA1-1 (Fig 1B). We then generated *in vitro*-transcribed truncated mutants of vtRNA1-1 to narrow down the region of the RNA important for RNP formation (S1A and S1B Fig). The band shift was markedly reduced by deleting the lower stem region of the RNA (Trunc.2), while it was only slightly affected by deleting the central loop region (Trunc.1) (S1C and S1D Fig).

Next, to gain insight into the putative protein binding partner of vtRNA1-1, we re-analyzed our previously identified vtRNA-RNPs dataset [11]. Using RAT-tagged vtRNAs for affinity purification in BL41 cells followed by LC-MS/MS, TRIM21 (Ro52/SSA1) turned out as one of the strongest candidates interacting with both vtRNA1-1 and vtRNA1-2. TRIM21, which belongs to tripartite motif-containing (TRIM) protein family with more than 80 E3 ubiquitin ligases, contains an N-terminal RBCC motif consisting of a RING domain, B-box, and coiled-coil domain that ubiquitylates their target proteins [17,18]. By their C-terminal PRY/SPRY domain, TRIM21 binds to and ubiquitylates target proteins, including immunoglobulin Fc [19]. Recent studies have also revealed that TRIM21 interacts with snoRNAs in human breast cancer cells [20].

To validate the possible interaction between vtRNA1-1 and TRIM21 also in HCC cells, we conducted RNA EMSA using extracts from vtRNA1-1 KO Huh7 cells with either depleted or overexpressed TRM21. The upshifted band intensity of vtRNA1-1 decreased upon TRIM21 knockdown (Fig 1B) and increased with overexpressing TRIM21 (Fig 1C), suggesting that TRIM21 is a binding partner of vtRNA1-1. This interaction was confirmed through formaldehyde crosslinking-immunoprecipitation (fCLIP) followed by RT-qPCR (Fig 1D). In vtRNA1-1 KO Huh7 cells, no RT-qPCR signal was detected, thus providing specificity to the setup. TRM21 immunoprecipitation did not pull-down snoRNA, thus hinting at a specific vtRNA1-1 RNP formation (Fig 1D). Removing the lower stem region of vtRNA1-1 resulted in a severe reduction in RNP formation as assessed by EMSA (S1E Fig). The remaining weak vtRNA1-1 band shift was, however, independent of TRM21 knockdown, indicating the crucial role of the stem region in the interaction with TRIM21. Given that the sequence and predicted structure in the stem region of all three human vtRNA1 paralogs are nearly identical, this result aligns with the previous LC-MS/MS analysis predicting that TRIM21 can interact with both vtRNA1-1 and vtRNA1-2 [11].

TRIM21 belongs to the C-IV subfamily, one of the elven TRIM protein subfamilies [21]. Notably, TRIM25, another member of this subfamily, was reported by others as a putative vtRNA1-1 binding protein [22]. Given the structural similarity of the C-terminal PRY/SPRY domain responsible for RNA interaction between TRIM25 and TRIM21 [23], we investigated the relationship between TRIM25 and vtRNA1-1 in more detail. Using fCLIP followed by RT-qPCR, we confirmed the interaction of TRIM25 with vtRNA1-1 in HCC cells (S1F Fig). Like TRIM21, the lower stem region of vtRNA1-1 is crucial for binding to TRIM25 (S1G Fig). Collectively, these findings suggest that vtRNA1-1 interacts with both TRIM21 and TRIM25 *in vivo* and *in vitro* via the lower stem region.

### TRIM21 and TRIM25 affect the stability of vtRNA1-1 in hepatocellular carcinoma cells

**Fig 2:**
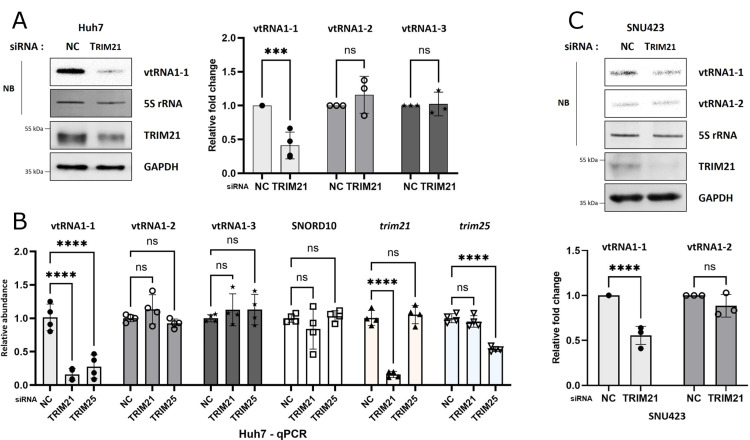
Depletion of TRIM21 or TRIM25 reduces vtRNA1-1 levels in hepatocellular carcinoma cells. (A) Northern blot analysis (NB) using the indicated probes (left, top), and immunoblot analysis using the indicated antibodies (left, bottom) in Huh7 cells. After transfecting Huh7 cells with the siRNA targeting TRIM21, total RNA and protein lysate were obtained and analyzed. NC denotes the negative control (mock transfection). 5S rRNA and GAPDH served as RNA and protein loading controls, respectively. The intensity of vtRNAs was normalized to that of 5S rRNA (right, n=3). Error bars indicate the standard deviation. ***p < 0.001, ns: non-significant (two-way ANOVA test). (B) RT-qPCR analysis using primers targeting the indicated RNAs (n=4). After transfecting Huh7 cells with the siRNAs either targeting TRIM21 or TRIM25, total RNA was extracted and analyzed by qPCR. Error bars indicate the standard deviation. ****p < 0.0001, ns: non-significant (two-way ANOVA test). (C) Same as in (A) but using the HCC cell line SNU423.

In order to elucidate the potential function of TRIM21 bound to vtRNAs, we investigated the levels of the three vtRNA1 paralogs after depleting TRIM21. In Huh7 cells, knockdown of TRIM21 led to a significant reduction in vtRNA1-1 levels (Figs 2A and 2B and S2A), while levels of the other vtRNAs remained unaffected (Figs 2B and S2A–C). Additionally, both nuclear and cytoplasmic vtRNA1-1 levels were reduced upon TRIM21 knockdown (S2D Fig). Transfection with a different TRIM21-targeted siRNA yielded identical results, excluding potential off-target effects of specific siRNA (S2E Fig). In other HCC cell lines, namely SNU423 and HepG2, TRIM21 depletion also reduced vtRNA1-1 levels, consistent with the observations in Huh7 cells (Figs 2C and S2F and S2G). To determine whether this vtRNA1-1 regulation is dependent on the cancer type, we also tested cervical cancer cells (HeLa). In contrast to liver cancer cells, TRIM21 knockdown did not affect vtRNA1-1 levels in HeLa cells (S2H Fig), suggesting that the effect of TRIM21 depletion on vtRNA1-1 might be cancer-type dependent. Overexpression of TRIM21 in the same set of cell lines had no effect on the vtRNA1-1 regardless of cancer type (S2I-K Fig)*,* indicating that endogenous TRIM21 levels are already saturating.

Similar to TRIM21, depleting TRIM25 also decreased vtRNA1-1 levels in HCC cells, without significantly affecting on other vtRNAs (Figs 2B and S2C and S3A–C), while TRIM25 overexpression showed no effect on vtRNA1-1 levels (S3D Fig). In a double knockdown system, we examined whether depleting both TRIM proteins had an additive effect on vtRNA1-1 levels. Compared to TRIM21 single knockdown, TRIM25 single knockdown led to a smaller reduction in vtRNA1-1, and the double knockdown of both TRIM proteins did not show any additive effect on vtRNA1-1 levels (S3E Fig). Additionally, we investigated the relevance of other TRIM protein family members for vtRNA1-1 stability. Knockdown of three different TRIM proteins, TRIM2, TRIM26, or TRIM65, all of which are known to interact with RNA, had no effect on vtRNA1-1 levels (S3F Fig).

**Fig 3:**
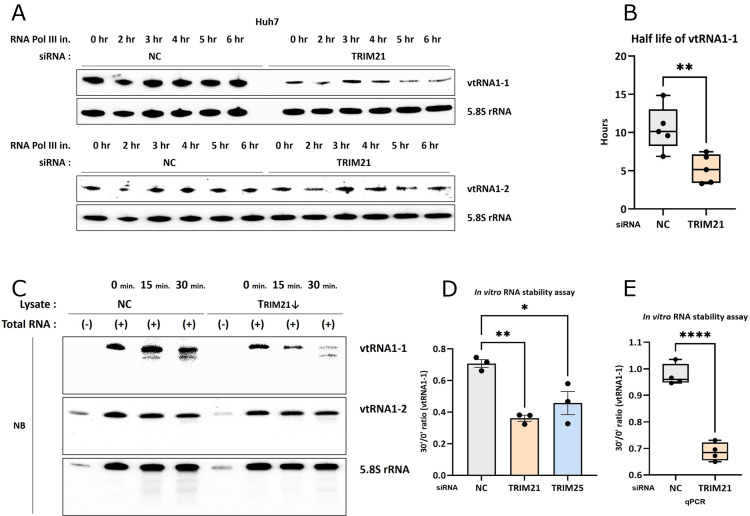
TRIM21 and TRIM25 regulates stability of vtRNA1-1. (A) RNA stability assay followed by northern blot analysis using the indicated probes. After transfecting Huh7 cells with the siRNA targeting TRIM21 and treating with RNA polymerase III inhibitor for the indicated times, total RNA was extracted and analyzed. NC denotes the negative control (mock transfection). 5.8S rRNA served as a loading control. (B) Half-life of vtRNA1-1 calculated from RT-qPCR analysis from the same set of total RNAs as in Fig 3A (n=5). Error bars indicate the standard deviation. **p < 0.01 (unpaired *t t*est) (C) *In vitro* RNA stability assay followed by northern blot analysis using the indicated probes. Total RNA was extracted from WT Huh7 cells, mixed with lysate obtained from vtRNA1-1 KO Huh7 cells transfected with the siRNA targeting TRIM21, and incubated for the indicated time periods. RNA extracted from the mixtures was analyzed. 5.8S rRNA served as a loading control (D) 30 min./0 min. ratio of vtRNA1-1 levels measured by the *in vitro* RNA stability assay followed by northern blot analysis (n=3, normalized to 5.8S rRNA). Total RNA was extracted from WT Huh7 cells and mixed with lysate obtained from vtRNA1-1 KO Huh7 cells transfected with the siRNAs either targeting TRIM21 or TRIM25. RNA extracted from the mixtures was analyzed. Error bars indicate the standard deviation. *p < 0.05, **p < 0.01, (one-way ANOVA test) (E) 30 min./0 min. ratio of vtRNA1-1 level measured by the *in vitro* RNA stability assay followed by RT-qPCR analysis (n=4, normalized to 18S rRNA). RNA extracted from the same set of mixtures as in Fig 3C was analyzed. Error bars indicate the standard deviation. ****p < 0.0001 (unpaired *t* test).

To clarify whether the decrease in vtRNA1-1 levels upon TRIM21 depletion is due to reduced transcription or diminished stability, we investigated the stability of vtRNA1-1 using both *in vivo* and *in vitro* RNA stability assay. Cells were treated with RNA polymerase III inhibitors to block *de novo* transcription of vtRNAs, in the absence or presence of TRIM21 RNAi, followed by northern blot and RT-qPCR analyses to assess the vtRNA levels. The rate of vtRNA1-1 degradation was faster in TRIM21-knockdown cells compared to control cells (Fig 3A, top), whereas that of vtRNA1-2 remained unaffected (Fig 3A, bottom). The *in vivo* RNA stability assay followed by RT-qPCR revealed a significantly shortened half-life of vtRNA1-1 upon TRIM21 depletion (Fig 3B). These findings were corroborated by an *in vitro* RNA stability assay showing that TRIM21-depleted lysates prepared from vtRNA1-1 KO cells degraded vtRNA1-1 much faster than control lysates, without affecting vtRNA1-2 (Figs 3C-E and S4A). These data demonstrated that TRIM21 affects vtRNA1-1 stability rather than modulating its abundance at the transcriptional level. Furthermore, TRIM25 knockdown similarly reduced the stability of vtRNA1-1. (Figs 3D and S4B-D). Thus, our findings suggest that TRIM21 and TRIM25, newly identified vtRNA binding proteins, are associated with the stabilization of vtRNA1-1 in HCC cells without affecting other vtRNA paralogs.

### The ‘UUAC’ sequence is crucial for the stability of vtRNA1-1

**Fig 4:**
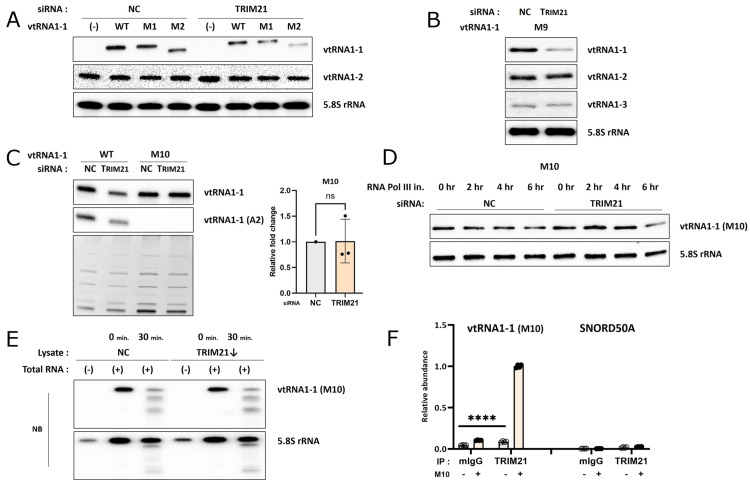
The ‘UUAC’ sequence is crucial for the stability of vtRNA1-1. (A and B) Northern blot analysis of the vtRNAs and vtRNA1-1 mutants (M1, M2, and M9) expressed in the vtRNA1-1 KO Huh7 cells after TRIM21 knockdown using the indicated probes. NC denotes the negative control (mock transfection). 5.8S rRNA served as a loading control. (C) Northern blot analysis of the vtRNA1-1 M10 mutant expressed in the Huh7 KO cell line using the indicated probes (left; the probe A2 was designed to solely detect WT vtRNA1-1). The EtBr-stained gel (on bottom) served as a loading control. The intensity of vtRNA1-1 M10 mutant was normalized to that of 5S rRNA (right, n=3). Error bars indicate the standard deviation. ns: non-significant (unpaired *t* test) (D) RNA stability assay followed by northern blot analysis using the indicated probes. After transfecting with the siRNA targeting TRIM21 and transiently expressing vtRNA1-1 M10 mutant, vtRNA1-1 KO Huh7 cells were treated with RNA polymerase III inhibitor for the indicated time periods. 5.8S rRNA served as loading control. (E) *In vitro* RNA stability assay followed by northern blot analysis using the indicated probes. Total RNA was extracted from Huh7 cells expressing the vtRNA1-1 M10 mutant, mixed with lysate obtained from vtRNA1-1 KO Huh7 cells transfected with the siRNA targeting TRIM21, and incubated for 0- or 30 min. RNA extracted from the mixtures was analyzed. 5.8S rRNA served as loading control. (F) fCLIP using the indicated antibodies (mIgG, TRIM21) and lysates obtained from cells either expressing vtRNA1-1 M10 mutant (+) or not (-). Co-immunoprecipitation was assessed by RT-qPCR using primers specific for vtRNA1-1 or SNORD50A. Error bars indicate the standard deviation. ****p < 0.0001 (two-way ANOVA test).

To determine the critical region of vtRNA1-1 for TRIM21-dependent stability, we created eleven mutants of vtRNA1-1 (S5A Fig). Although the lower stem region appeared to be involved in the interaction with TRIM21 and TRIM25 (S1E and S1G Fig), mutations were generated in the central loop or the upper stem region of the vtRNA1-1. The rationale behind this design was influenced by our findings that despite nearly identical sequences in the lower stem region of all vtRNAs, the levels of vtRNA1-2 and vtRNA1-3 were not affected by TRIM proteins (Figs 2A and 2B and S3A). M1 to M9 mutants, carrying mutations in the central loop region, were expressed in Huh 7 vtRNA1-1 KO cells. All of these mutants showed reduced cellular levels upon TRIM21 knockdown, like the WT vtRNA1-1 (Figs 4A and 4B and S5B-D). In contrast, however, the M10 mutant, harboring four consecutive base changes at positions 21 to 24 in the upper stem (Fig 1A, red circle), was unaffected by TRIM21 knockdown (Figs 4C and S5E), while the M11 mutant, harboring two substituted nucleotides within the different parts of the upper stem, still decreased (S5F Fig, left). As with TRIM21, TRIM25 knockdown also showed no effect on the M10 mutant (S5F Fig, right), while all other mutants decreased (S5F-J Fig). Both *in vivo* and *in vitro* stability assays demonstrated that the M10 mutant of vtRNA1-1 was unaffected by TRIM21 depletion (Figs 4D and 4E and S5K), and TRIM25 depletion (S5L Fig). Thus, we concluded that the ‘UUAC’ sequence (positions 21-24) in the upper stem region of vtRNA1-1 is crucial for the TRIM21-dependent vtRNA1-1 stability.

We further evaluated whether the M10 mutant lost its capability to bind to TRIM21. Interestingly, the M10 mutant of vtRNA1-1 still bound to TRIM21 *in vivo* in a similar fashion and specificity as the WT vtRNA1-1 as assessed by the fCLIP (Fig 4F). Moreover, the M10 mutant retained its interacting potential with proteins in cell lysate *in vitro*, and its binding affinity was diminished upon TRIM21 knockdown (S5M Fig). These results suggested that the ‘UUAC’ sequence of vtRNA1-1 is not directly implicated in the interaction with TRIM21, despite its apparently critical role in stability regulation. Therefore, additional factors beyond vtRNA1-1/TRIM21 RNP formation are required to fine-tune cellular vtRNA1-1 levels.

### NSUN2-mediated methylation at C69 of vtRNA1-1 matters for stability

**Fig 5:**
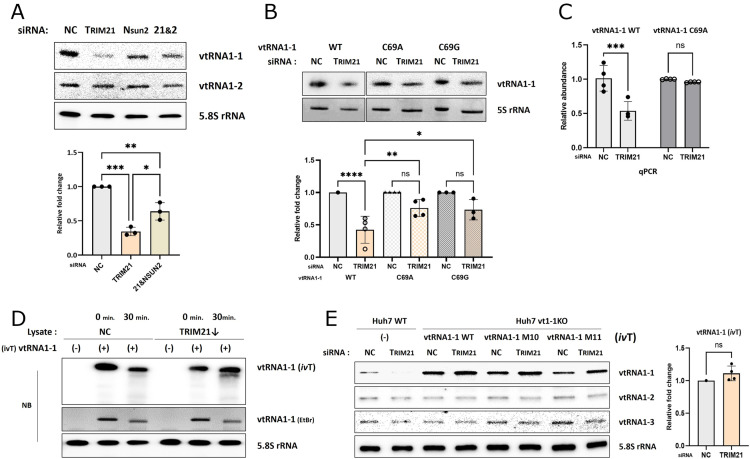
NSUN2-mediated methylation at C69 of vtRNA1-1 is implicated in the stability regulation. (A) Northern blot analysis using the indicated probes (top). After transfecting WT Huh7 cells with the siRNAs either targeting TRIM21, NSUN2, or both simultaneously (21&2), total RNA was extracted and analyzed. NC denotes the negative control (mock transfection). 5.8S rRNA served as loading control. The intensity of vtRNA1-1 was normalized to that of 5.8S rRNA (bottom, n=3). Error bars indicate the standard deviation. *p < 0.05, **p < 0.01, ***p < 0.001 (one-way ANOVA test). (B) Northern blot analysis of the vtRNA1-1 C69A and C69G mutants expressed in the Huh7 vtRNA1-1 KO cells after TRIM21 knockdown using the indicated probes. 5S rRNA served as loading control. The ratio of normalized vtRNA1-1 intensity to that of 5S rRNA in TRIM21 knockdown cells and in control cells was calculated (right, n=3). Error bars indicate the standard deviation. *p < 0.05, **p < 0.01, ****p < 0.0001, ns: non-significant (one-way ANOVA test). (C) RT-qPCR analysis of vtRNA1-1 WT and C69A mutant expressed in the Huh7 vtRNA1-1 KO cells after TRIM21 knockdown (n=4). Error bars indicate the standard deviation. ***p < 0.001, ns: non-significant (two-way ANOVA test) (D) *In vitro* RNA stability assay followed by northern blot analysis using the indicated probes. *In vitro*-transcribed (*iv*T) vtRNA1-1 was mixed with lysate obtained from vtRNA1-1 KO Huh7 cells transfected with the siRNA targeting TRIM21, and incubated for 0- or 30 min. RNA extracted from the mixtures was analyzed. 5.8S rRNA served as loading control. (E) Northern blot analysis using the indicated probes (left). After transfecting WT or vtRNA1-1 KO Huh7 cells with the siRNAs either targeting TRIM21 or TRIM25 and with the *in vitro*-transcribed (*ivT*) vtRNA1-1 WT, M10, and M11 mutants, total RNA was extracted and analyzed. 5.8S rRNA served as loading control. The intensity of *in vitro*-transcribed vtRNA1-1 was normalized to that of 5.8S rRNA (right, n=3). Error bars indicate the standard deviation. ns: non-significant (unpaired *t* test).

To find such additional factors for vtRNA1-1 stability, we investigated post-transcriptional modifications on this ncRNA. In line with numerous other short ncRNAs such as tRNA or rRNA, vtRNAs also undergo post-transcriptional modifications [15]. The best characterized modification of vtRNA1-1 thus far is C5 methylation at cytosine 69 mediated by NSUN2, which governs the production of svRNA [16]. We delved into the significance of 5-methylcytosine of vtRNA1-1 for its stability regulation. Co-transfecting cells with siRNAs for NSUN2 and TRIM21 rescues vtRNA1-1 levels compared to the TRIM21 single knockdown (Figs 5A and S6A and S6B), suggesting the involvement of m^5^C69 in TRIM21-dependent vtRNA1-1 stabilization. To provide direct evidence of the relationship between the methylation status and stability of vtRNA1-1, we created two different point mutants of vtRNA1-1, C69A and C69G (Figs 1A, blue circle, and S6C), which cannot be methylated by NSUN2. Since C69 of vtRNA1-1 is located within the internal promoter region of the RNA known as B-box, which is essential for RNA polymerase III-mediated transcription, we first validated their robust expression in Huh7 cells (S6D Fig). Both the C69A and C69G mutants became more resistant to degradation upon TRIM21 or TRIM25 depletion compared to WT vtRNA1-1 (Figs 5B and 5C and S6E). Notably, the stability of C69A vtRNA1-1, assessed by *in vitro* RNA stability assay, was unaffected by TRIM21 depletion (S6F Fig). These findings suggest that unmethylated vtRNA1-1 is more stable under TRIM21-depleted condition. In contrast, m^5^C69 containing vtRNA1-1 is prone for degradation when cellular TRIM21 levels are reduced.

To further evaluate the hypothesis that the m^5^C69 modification of vtRNA1-1 is crucial for its stability regulation, we utilized *in vitro*-transcribed vtRNA1-1 which lacks modifications. *In vitro* RNA stability assay with *in vitro*-transcribed vtRNA1-1 clarified that TRIM21 levels had little effect on the stability of unmodified vtRNA1-1 (Fig 5D). Moreover, the levels of *in vitro*-transcribed vtRNA1-1 transfected into cells remained unaltered upon both TRIM21 and TRIM25 knockdown, whereas endogenous vtRNA1-1 showed a massive reduction under the same condition (Figs 5E and S6G). Overall, these findings indicated that NSUN2-mediated methylation at C69 of vtRNA1-1 is closely related to the stability of the RNA, which is controlled by TRIM21 and TRIM25 in HCC cells. Methylated vtRNA1-1 at C69 was susceptible to degradation under TRIM21-depleted condition.

### vtRNA1-1 stability is influenced by the TRIM21-RBCK1-TRIM25 axis

**Fig 6:**
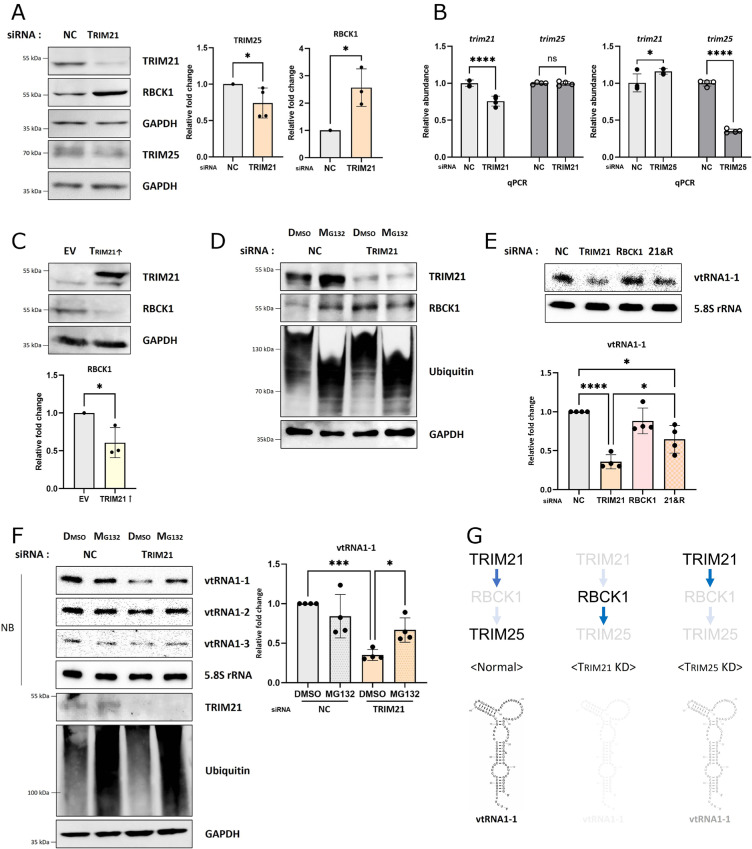
The stability of vtRNA1-1 is influenced by the TRIM21-RBCK1-TRIM25 axis. (A) Immunoblot analysis using the indicated antibodies (left). After transfecting WT Huh7 cells with the siRNA targeting TRIM21, lysates were obtained and analyzed. NC denotes the negative control (mock transfection). GAPDH served as loading control. The intensity of indicated proteins was normalized to that of GAPDH (right, n=4 for TRIM25, and n=3 for RBCK1). Error bars indicate the standard deviation. *p < 0.05 (unpired *t t*est) (B) RT-qPCR analysis of *trim21* and *trim25* mRNA (n=4) after transfecting WT Huh7 cells with the siRNAs either targeting TRIM21 or TRIM25. Error bars indicate the standard deviation. *p < 0.05, ****p < 0.0001, ns: non-significant (two-way ANOVA test) (C) Immunoblot analysis using the indicated antibodies (left). After overexpressing TRIM21, lysates were obtained and analyzed. EV denotes the empty vector (mock transfection). GAPDH served as loading control. The intensity of RBCK1 was normalized to that of GAPDH (right, n=3). Error bars indicate the standard deviation. *p < 0.05 (unpaired test) (D) Immunoblot analysis using the indicated antibodies. After transfecting WT Huh7 cells with the siRNA targeting TRIM21 and treating with proteasomal inhibitor (MG132) or not (DMSO), lysates were obtained and analyzed. GAPDH served as a loading control. (E) Northern blot analysis using the indicated probes (top). After transfecting WT Huh7 cells with the siRNAs either targeting TRIM21, RBCK1, or both simultaneously (21&R), total RNA was extracted and analyzed. 5.8S rRNA served as loading control. The intensity of vtRNA1-1 was normalized to that of 5.8S rRNA (bottom, n=4). Error bars indicate the standard deviation. *p < 0.05, ****p < 0.0001 (one-way ANOVAtest) (F) Northern blot analysis using the indicated probes (left, top), and immunoblot analysis using the indicated antibodies (left, bottom). After transfecting WT Huh7 cells with the siRNA targeting TRIM21 and treating with proteasomal inhibitor (MG132) or not (DMSO), total RNA and lysate were obtained and analyzed. 5.8S rRNA served as loading control. The intensity of vtRNA1-1 was normalized to that of 5.8S rRNA (right, n=4). Error bars indicate the standard deviation. *p < 0.05, ***p < 0.001 (one-way ANOVA test) (G) Schematic model for the regulatory process of vtRNA1-1 stability mediated by the TRIM21-RBCK1-TRIM25 axis. Under normal conditions, TRIM21 mediates the proteasomal degradation of RBCK1, preventing TRIM25 degradation, thereby stabilizing vtRNA1-1 through the functions of TRIM21 and TRIM25 (left). When TRIM21 is depleted, undegraded RBCK1 induces TRIM25 degradation. This simultaneous lack of both TRIM21 and TRIM25 leads to a reduction in vtRNA1-1 stability (middle). Since TRIM25 knockdown shows no effect on TRIM21 levels, TRIM21 partially maintains vtRNA1-1 stability in the absence of TRIM25 (right).

To further understand the regulatory process of vtRNA1-1 stabilization, we investigated the relationship between TRIM21 and TRIM25 in HCC cells. Interestingly, we found that TRIM25 protein levels were reduced upon TRIM21 depletion (Figs 6A and S7A), whereas TRIM25 knockdown had no significant effect on TRIM21 protein levels (S7B and S7C Fig). These observations suggest that TRIM21 acts upstream in regulating TRIM25 levels, consistent with our previous data showing that TRIM21 knockdown led to a stronger reduction in vtRNA1-1 than TRIM25 knockdown (S3E Fig). Since the *tirm25* mRNA levels were unaffected by TRIM21 knockdown (Fig 6B), this indicated that the reduction in TRIM25 due to TRIM21 depletion is a post-transcriptional effect. Inn et al. reported that TRIM25 degradation via the proteasomal degradation system is controlled by the linear ubiquitin chain assembly complex (LUBAC) [24], consisting of two E3 ligases, RBCK1 (HOIL-1L) and HOIP. Consistent with the findings that increased RBCK1 reduces TRIM25 levels [24], RBCK1 was upregulated in TRIM21 knockdown cells to promote TRIM25 degradation (Figs 6A and S7A and S7D), while TRIM21 overexpression reduced RBCK1 levels (Fig 6C). Inhibition of the proteasomal degradation led to RBCK1 accumulation under normal conditions (Fig 6D). However, after transfecting cells with TRIM21 siRNA, accumulated RBCK1 was observed even in the absence of proteasomal degradation inhibitor (Fig 6D), indicating RBCK1 is resistant to degradation via the proteasomal system in TRIM21-depleted cells. Therefore, we concluded that TRIM21 stabilizes TRIM25 by inducing the degradation of RBCK1, the E3-ligase responsible for ubiquitylating TRIM25.

To gain insight into the function of RBCK1 in the context of vtRNA1-1 stability, we co-transfected cells with the siRNAs targeting TRIM21 and RBCK1. While RBCK1 knockdown did not affect vtRNA1-1 levels (S7E Fig), double knockdown of TRIM21 and RBCK1 rescued the vtRNA1-1, mitigating the effect of TRIM21 depletion (Fig 6E). The inhibition of proteasomal degradation also rescued the reduced vtRNA1-1 levels in TRIM21-depleted HCC cells (Figs 6F and S7F), suggesting that TRIM25, not degraded via RBCK1-mediated proteasomal degradation, partially maintains vtRNA1-1 stability in the absence of TRIM21. In contrast, inhibiting proteasomal degradation did not rescue the reduced vtRNA1-1 in TRIM25-depleted cells (S7G Fig). These data indicate that the regulatory axis consisting of three E3 ligases, TRIM21, RBCK1, and TRIM25, controls the stability of vtRNA1-1 in HCC cells (Fig 6G).

### TRIM21-dependent vtRNA1-1 stabilization is crucial for pro-survival characteristics in HCC cells

It has been reported that vtRNA1-1 modulates pro-survival characteristics in many cancer cell lines, deriving from lymphoma, lung cancer, and cervical cancer [7,10,11]. Our group previously identified the influence of vtRNA1-1 on HCC proliferation using ectopic vtRNA1-1 expression in the vtRNA1-1 KO Huh7 cells [12]*.* To corroborate the pro-survival function of vtRNA1-1 in WT Huh7 cells, we measured the proliferation rate in the cells overexpressing vtRNA1-1 (S8A Fig). Overexpressing vtRNA1-1 increased the proliferation rate (S8B and S8C Fig), confirming that vtRNA1-1 is a proliferative factor in HCC cells.

**Fig 7:**
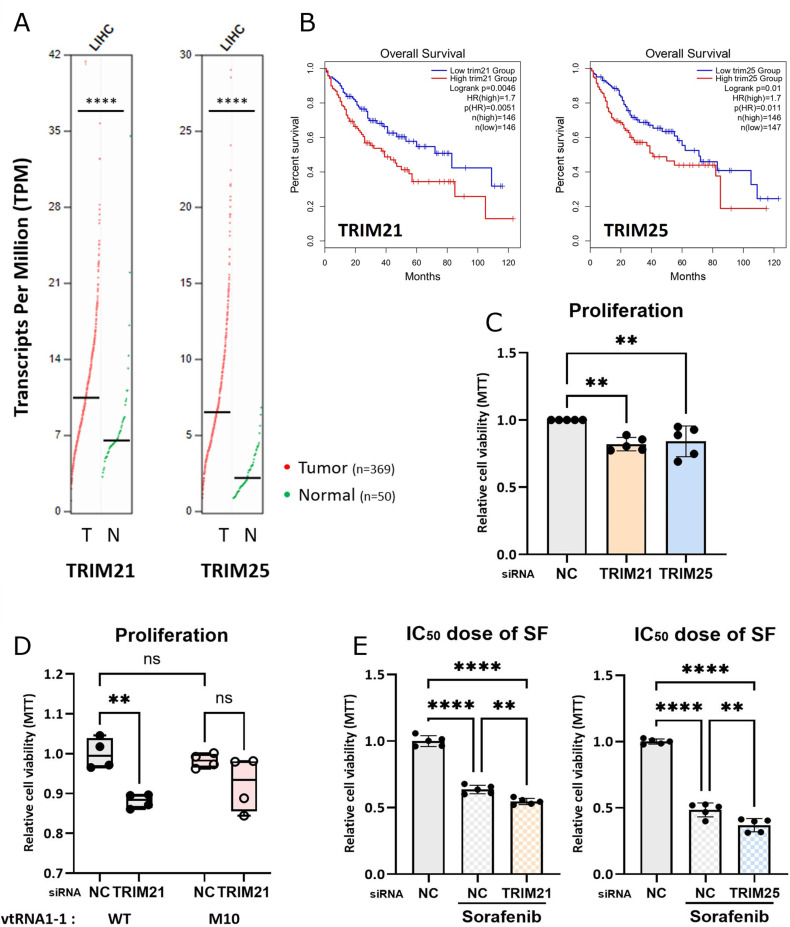
vtRNA1-1 stability regulation is crucial for pro-survival characteristics in HCC cells. (A) Expressions of TRIM21 and TRIM25 in LIHC (liver hepatocellular carcinoma) tissues (T, n=369) comparing with normal tissues (N, n=50) presented by TCGA database were analyzed using GEPIA analysis program (|log_2_FC| cutoff=0.5489). (B) Survival analysis of liver hepatocellular carcinoma patients based on the expression status of the indicated single gene. Dataset was presented by TCGA database and analyzed using GEPIA analysis program (HR: hazards ratio). (C) Relative proliferation rates of Huh7 cells after transfecting with the siRNAs either targeting TRIM21 or TRIM25 were measured by the MTT assay (n=5). NC denotes the negative control (mock transfection). Error bars indicate the standard deviation. **p < 0.01 (one-way ANOVA test) (D) Relative proliferation rates of vtRNA1-1 KO Huh7 cells transiently expressing vtRNA1-1 WT or M10 after TRIM21 knockdown were measured by MTT assay (n=4). Error bars indicate the standard deviation. **p < 0.01, ns: non-significant (two-way ANOVA test) (E) Relative cell viabilities of Huh7 WT or vtRNA1-1 KO cells after transfecting with the siRNAs either targeting TRIM21 or TRIM25 in the presence of an IC_50_ dose of sorafenib (24 hours, 12 µM) were measured by the MTT assay (n=5). Values were normalized to that of untreated cells. Error bars indicate the standard deviation. **p < 0.01, ****p < 0.0001 (one-way ANOVA test).

To gain insight into the interconnection between TRM21/TRIM25 levels, vtRNA1-1 abundance, and pro-survival characteristics of HCC cells, we analyzed the expression of TRIM21 and TRIM25 using the GEPIA analysis program, with data provided by the TCGA dataset [25,26]. Compared with their normal counterparts, mRNA levels of both *trim21* and *trim25* are upregulated in liver cancer tissues (Fig 7A), consistent with previous reports [27–30]. In line with these findings, comparing overall patient survival between high and low expression groups of TRIM21 and TRIM25 suggests that upregulation of these TRIM proteins correlates with tumor progression (Figs 7B and S8D). In line with our expectations, depleting TRIM21 or TRIM25, which lowers vtRNA1-1 levels (Figs 2A and 2B and S3A), reduced the proliferation rate in HCC cells (Figs 7C and S8E). Since elevated expressions of both TRIM proteins are known to contribute to the tumor progression of HCC and poor prognosis via vtRNA1-1-independent mechanisms [27,29,31], we investigated the influence of TRIM21/TRIM25-dependent vtRNA1-1 stability on the proliferation of HCC cells using the vtRNA1-1 M10 mutant, which is resistant to degradation upon TRIM21 or TRIM25 knockdown. Indeed, the proliferation rate of cells expressing the M10 mutant did not significantly change upon TRIM21 knockdown, while that of WT vtRNA1-1-expressed cells was markedly reduced (Fig 7D). This indicates that TRIM21 regulates proliferation of HCC cells by modulating vtRNA1-1 stability. Another important feature of vtRNA1-1 deficiency in HCC cells is the increased cytotoxicity of sorafenib, the first-line treatment option for liver cancer [12]. TRIM21- or TRIM25-depleted HCC cells treated with an IC_50_ dose of sorafenib (S8F Fig) were less viable compared to control cells (Figs 7E and S8G and S8H). Interestingly, the increased cytotoxicity of sorafenib upon TRIM21 knockdown was not observed in vtRNA1-1 KO cells expressing the M10 mutant, (S8I and S8J Fig). These data indicated that stability regulation of vtRNA1-1 is important for the cytotoxicity of sorafenib.

In summary, our findings elucidate the novel stabilization mechanism of vtRNA1-1 in HCC cells, involving two newly identified vtRNA1-1 interacting proteins, TRIM21 and TRIM25. Additionally, the methylation status of vtRNA1-1 at C69, mediated by NSUN2, plays a critical role in modulating TRIM21/TRIM25-dependent vtRNA1-1 stability. We also demonstrated that TRIM21 and TRIM25 are crucial for the pro-survival characteristics of HCC cells through modulating vtRNA1-1 stability.

## Discussion

It is no longer surprising that short ncRNAs play significant roles in regulating a multitude of cellular processes. In the past few decades, research has identified a diverse array of regulatory ncRNAs and elucidated their functions. Discovered in the mid-1980s [3], the short non-coding vtRNA has since been verified to regulate a variety of intracellular functions, including proliferation, apoptosis, and autophagy [4,7,10–13,16]. Specifically, a comprehensive and in-depth understanding of vtRNA1-1, which has pro-survival characteristics in many cancer cell lines of different tissue origin, could pave the way to developing novel therapeutic strategies or diagnostic approaches. Despite their importance, regulatory mechanisms to control vtRNA levels in cells remained elusive. Here, we describe how vtRNA1-1 stability and cellular abundance are modulated by its interaction with TRIM21 and TRIM25. We re-analyzed our initial vtRNA-RNP dataset (Bracher et al., 2020 [11]) and identified the interaction between vtRNA1-1 and these TRIM proteins in HCC cells, Furthermore, we revealed the importance of NSUN2-mediated methylation at C69 of vtRNA1-1 for its stability. This regulation appears to be cancer-type dependent: it is observed in HCC cells but not in cervical cancer cells (Fig 2). As the function of vtRNA1-1 also differs slightly between these two types of cancer cells [10–12], our findings suggest that the properties of vtRNA1-1 may vary by cancer type.

In this study, we identified novel factors contributing to the stability of vtRNA1-1 in HCC cells. However, further research is needed to fully comprehend the precise mechanism of vtRNA1-1 stabilization. Even though vtRNA1-1 clearly interacts with TRIM21 or TRIM25 (Fig 1 and S1 Fig), it remains unclear whether the interactions are directly or indirectly mediated by other so far unidentified proteins or nucleic acids. Moreover, it is necessary to elucidate how TRIM21 and TRIM25 exactly regulate vtRNA1-1 stability. Thus far, nothing is known about a direct role of TRIM21 or TRIM25 to RNA degradation, as no RNase activities have been described for these two TRIM proteins.

The interactions between vtRNA1-1 and TRIM21 or TRIM25 are not the sole factors regulating vtRNA1-1 stability in HCC cells. The nucleotide sequence of the lower stem responsible for the interaction of vtRNA1-1 with these TRIM proteins (Fig 1A), is nearly identical to that of vtRNA1-2 and vtRNA1-3. This indicates that TRIM21 and TRIM25 are putative interacting partners of all three paralogs of vtRNA1. However, unlike vtRNA1-1, neither vtRNA1-2 nor vtRNA1-3 are influenced by TRIM21 or TRIM25 levels in HCC cells (Fig 2). The retained interaction between the vtRNA1-1 M10 mutant, harboring base changes within the crucial region for the stability regulation, and TRIM21 also strongly supports that additional factors are required to govern the vtRNA1-1 stability process. Thus, we presented NSUN2-mediated methylation at C69 as another factor associated with vtRNA1-1 stabilization (Fig 5). The methylation status of vtRNA1-1 is important for its stability regulated by the TRIM proteins. The stability of unmethylated vtRNA1-1 remains unaffected upon depleting TRIM21 or TRIM25 in HCC cells. However, it is still needed to elucidate the relationship between the methylation status of vtRNA1-1 and the TRIM proteins in the perspective of stability regulation. It might be worthwhile to explore how vtRNA1-1 interacts with TRIM proteins depending on its methylation status. Investigating the involvement of the ‘UUAC’ sequence, mutated in M10 mutant (Fig 1A), and m^5^C69 of vtRNA1-1 could be another valuable approach to unveil the precise mechanism of vtRNA1-1 stabilization. Collectively, an in-depth understanding of the relationship between all the factors involved in vtRNA1-1 stability, such as the methylation status, the ‘UUAC’ sequence, or the as of yet unidentified RNase, is still needed.

An additional notable finding about vtRNAs, as revealed to date, is its involvement in innate immunity induced by viral infection. During infection with influenza A virus (IAV), or Epstein-Barr virus (EBV), the expression of vtRNAs increases significantly, enhancing viral establishment by inhibiting PKR-mediated innate immunity (IAV) [32], or suppressing apoptosis (EBV) [10]. Interestingly, the two TRIM proteins regulating the vtRNA1-1 stability are also closely related to immune responses against viral infection [17,18,21,33]. The dynamics of the ubiquitination status of both host and viral proteins play various roles in viral infection and innate immune responses [34–37]. As E3-ligases mediating ubiquitination of target proteins, the functions of TRIM21 and TRIM25 on the innate immune response are well-established. For example, TRIM21 regulates interferon signaling by interacting with several proteins such as MAVS, a pattern recognition receptor, DDX41, an intracellular dsDNA sensor recognizing viral DNA, and IFN-inducible protein 35 (IFI35) [38–40]. As an intracellular antibody receptor, TRIM21 recognizes internalized antibody-coated viruses by binding to the constant Fc region of antibodies, facilitating their ubiquitination and degradation via the proteasomal degradation system [41]. Moreover, it has been reported that TRIM21 interacts with virus-encoded proteins such as hepatitis B virus (HBV) DNA polymerase or HBx protein, promoting its degradation to prevent viral DNA replication [42,43]. TRIM25 is also known to play a significant role in the innate immune responses. In response to RNA viruses, TRIM25 mediates the ubiquitination of RIG-I, a pivotal cytosolic RNA sensor, leading to the activation of innate immune responses [44]. In addition to RIG-I, TRIM25 is related to the activation of NF-kB by inducing MDA5, a different type of pattern recognition receptor (PRR) detecting dsRNA in the cytoplasm [45]. Considering these observations and the interaction between vtRNA1-1 and TRIM21/25 we revealed here, it can be inferred that the regulation of vtRNA1-1 stability could also be implicated in modulating immune responses against viral infection. Therefore, exploring how their relationship influences innate immunity could be an intriguing topic to find novel antiviral strategies. Furthermore, elucidating the effects of vtRNA1-1 on the function of TRIM proteins on innate immunity could be considered as another attractive topic.

TRIM proteins are involved not only in regulating innate immune responses but also in a wide range of cellular processes, including tumorigenesis and cancer development [46]. Elevated TRIM25 is associated with the progression of several types of cancer, and its upregulation correlates with poor prognosis in HCC, breast cancer, and glioma [27,47,48]. Emerging evidence also suggests the involvement of TRIM21 in tumorigenesis, with a dual function in either promoting or suppressing cancer progression depending on cancer types and context [49]. In this study, we demonstrated a novel crosstalk between three E3 ligases; TRIM21, RBCK1, and TRIM25 (Fig 6). Although it has not been established whether TRIM21 directly ubiquitylates RBCK1, we verified that TRIM21 promotes the degradation of RBCK1 through the proteasomal system, thereby stabilizing TRIM25. Since reducing TRIM21 leads to a decrease in TRIM25 through RBCK1-mediated degradation, the effect of TRIM21 on stabilizing vtRNA1-1 is more significant than that of TRIM25. Given that elevated expressions of vtRNA1-1 and both TRIM proteins have been observed in HCC (Fig 7A) [7,27,29,30], a deeper examination of this TRIM21-RBCK1-TRIM25 axis in the perspective of vtRNA1-1 stability regulation could uncover underlying mechanisms involved in liver cancer progression to acquire pro-survival characteristics. Moreover, conducting *in vivo* studies could help clarify the functional relevance of our findings in tumorigenesis, as this study was primarily done *in cellulo* using HCC cell lines. The elevated TRIM21 and TRIM25 levels in liver cancer patients (Fig 7A) and the increased hazard ratio in patient groups with high TRIM21 and TRIM25 expression (Fig 7B) indicates the association of these TRIM proteins and vtRNA1-1 levels with tumor progression.

In conclusion, our study identified so far unknown novel mechanisms regulating vtRNA1-1 stability mediated by two TRIM proteins and the methylation status of vtRNA1-1 in HCC cells. vtRNA1-1 is a remarkably intriguing short ncRNA with implications for cancer and innate immunity. Recent research showed that vtRNA1-1 is a pro-survival factor contributing to cell proliferation, apoptosis, autophagy, and drug resistance [7]. Based on these insights, our investigation into the regulation of vtRNA1-1 stability via the TRIM21-RBCK1-TRIM25 axis and NSUN2-mediated methylation provides novel perspectives for developing effective anticancer and antiviral strategies.

## Methods

### Cell culture, transfection and treatments

The human hepatocellular carcinoma cell line Huh7 WT and *VTRNA1-1* KO (provided by Matthias Hentze; EMBL, Heidelberg, Germany), SNU423 and HepG2 (provided by Deborah Stroka; UniBe, Bern, Switzerland) and the human cervical cancer cell line HeLa (ATCC-CCL-2) were cultured in DMEM/F-12 (Gibco, 21331046) supplemented with 10% FBS (Gibco, 10082147) and 1x Pen-Strep glutamine (Gibco, 10378016). For reverse transfection of siRNA, Lipofectamine RNAiMAX (Invitrogen, 13778075) was used according to the manufacturer`s recommendations. For plasmid transfection or *in vitro*-transcribed RNA transfection, Lipofectamine 3000 (Invitrogen, L3000015) was used according to the manufacturer`s recommendations. Sorafenib tosylate (MedChemExpress, HY-10201A) was used from 10 to 14 μM for 24 hours. MG132 (Selleckchem, S2619) was used at 2 μM for 6 hours.

### Immunoblot and immunoprecipitation

Cells were harvested and lysed with lysis buffer (20 mM Tris-Cl pH 7.5, 150 mM NaCl, 2.5 mM MgCl_2_, 1% Triton X-100 (Sigma-Aldrich, T8787), 0.25% Na-deoxycholate (Sigma-Aldrich, D6750)), containing protease inhibitor cocktail (Sigma-Aldrich, 11697498001) and phosphatase inhibitor (Thermo Scientific, A32957) for 30 min on ice. The supernatants were collected after centrifugation at 16 000 ×g for 10 min at 4°C, and protein concentrations were determined using BCA protein assay kits (Thermo Scientific,23225). Whole protein lysates were boiled with 4× NuPage sample buffer (Invitrogen, NP0007), separated by SDS-PAGE gel and transferred to a 0.45 µm nitrocellulose membrane for immunoblotting. Immunoblotting was performed following standard protocols using the indicated antibodies. All quantified immunoblot signals were normalized to the band intensities of the GAPDH. For immunoprecipitation, at least 0.5 milligram of whole cell lysate were incubated with indicated primary antibodies (1:100 ratio) for 2-4 hours at 4°C with mixing and combined protein A/G magnetic beads (Thermo Scientific, 88802) with lysate-antibody mixtures for overnight at 4°C with mixing. After the overnight incubation, washing and elution were performed according to the manufacturer`s recommendations.

### RNA extraction, reverse transcription-PCR (RT-PCR) and quantitative real-time PCR (qPCR)

Total RNA was extracted from the harvested cells using TRI-reagent (Zymo Research, R2050-1-200) according to manufacturer’s recommendations. Reverse transcription of total RNA was performed using SuperScript IV One-Step RT-PCR System (Invitrogen, 18090010), and random hexamers according to the manufacturer’s recommendations. qPCR reactions were performed using Rotor Gene-Q (QIAGEN), and analyses were conducted in Robotics software (QIAGEN) according to the manufacturer’s instructions.

### Northern blot

4-6 µg of total RNA was separated on a denaturing polyacrylamide gel (7 M Urea, 1x TBE buffer), transferred to a nylon membrane (Amersham Hybond N^+^; GE Healthcare, RPN203B), UV-crosslinked, and probed with 5′-^32^P-end labeled indicated antisense DNA probes. Signals were exposed to a phosphor screen and quantified using a Typhoon FLA1000 phosphorimager and ImageQuantTL software. All quantified northern blot signals were normalized to the band intensities of the 5.8S rRNA or EtBr-stained 5S rRNA.

### RNA stability assay

For *in vivo* RNA stability assay, cells were treated with an RNA polymerase III inhibitor and incubated for indicated times in the incubator. The RNA levels extracted from the harvested cells were measured by northern blot or RT-qPCR as described above. For *in vitro* RNA stability assay, 30-50 µg of whole cell lysate obtained from the vtRNA1-1 KO Huh7 cells and 8-10 µg of total RNA extracted from indicated cells or 3-5 µg of *in vitro* transcribed RNA were mixed and incubated for indicated times at 37°C. RNA levels extracted from the mixture were measured by northern blot or RT-qPCR.

### Formaldehyde cross-linking immunoprecipitation (fCLIP)

fCLIP was performed as described in Kim et al. [50]. After cell growth, media was removed, and cells were washed with PBS. Formaldehyde solution (0.1%, diluted in PBS) was added for crosslinking, and incubated for 10 min at RT with gently mixing every 2 min. Glycine was added (150 mM of final concentration) for quenching and incubated at for 10 min RT with gently mixing every 2 min. After washing with PBS, the cells were harvested. Using the harvested cells, immunoprecipitation was performed as described above with indicated antibodies, and RNA extracted from the IP elute was analyzed by RT-qPCR.

### *In vitro* transcription

Wild type and truncated mutants of vtRNA1-1 were transcribed from DNA templates. DNA primers containing T7 promoter sequence were extended to form DNA templates, and the templates were amplified by PCR. After purifying PCR products with Wizard SV Gel and PCR Clean-Up (Promega, A9282), RNAs were transcribed by T7 polymerase with 7 hours incubation at 37°C. RNA transcripts were purified by size exclusion chromatograph using G-25 sephadex (Sigma-Aldrich, G2580).

### RNA EMSA

RNA EMSA was performed as described in Raad et al. [51]. *In vitro*-transcribed RNAs were dephosphorylated by CIP (NEB, M0525) according to manufacturer’s recommendations, and purified using phenol-chloroform and ethanol. For 5`-end labeling, PNK (NEB, M0201) was used according to manufacturer’s recommendations. 5`-end labelled RNAs were heated for 3 min at 85°C for denaturation and cooled down for 10 min at RT. RNAs (30 nM of final concentration) were incubated with indicated lysates (5-50 µg) and with binding buffer (100 mM Tris-Cl pH7.5, 100 mM MgCl_2_, 500 mM, KCl, 0.05 mM PMSF, 5% glycerol, 5 mM DTT, 0.1 u/µl RNasin, 40 µg/ml yeast tRNA, 1 mg/ml BSA, 0.1% NP-40) for 30 min at 37°C. After mixing with RNA dye, reaction mixtures were loaded on a 6% native acrylamide gel (6% acrylamide, 2.5% glycerol, 0.5X TBE). After 100 min running at 200 V, the gels were wrapped in plastic foil, then exposing to a screen at -20°C.

### Cell proliferation and viability assay

Cell proliferation was measured by MTT assay or cell counting. To assess cell viability, metabolic activity was measured using MTT. After incubating cells with indicated nucleic acids or drug at different time point, MTT solution in growth media was added and incubated at 37°C for 2 hours. The media was replaced by DMSO to dissolve formazan crystals and incubated 10 minutes protected from light. Absorbance was measured at 570 nm using a Tecan Infinite M1000Pro plate redear. To assess proliferation rate by cell counting, automated cell counter was used after mixing with trypan blue.

## Supporting information

S1 Fig (A) Sequences of *in vitro*-transcribed vtRNA1-1 WT and truncated mutants (Trunc.1 and Trunc.2). (B) EtBr-stained gel and northern blot analysis using the indicated probes detecting in vitro-transcribed vtRNA1-1 WT and mutants. (C and D) EMSA of radiolabeled vtRNA1-1 WT or truncated mutants with increasing concentration of cell lysate obtained from vtRNA1-1 KO Huh7 cells. (E) EMSA of radiolabeled vtRNA1-1 WT or truncated mutant (Trunc.2) with increasing concentration of cell lysate obtained from control or TRIM21 siRNAs-transfected vtRNA1-1 KO Huh7 cells (top). The arrow marks the upshifted RNP. (F) fCLIP using the indicated antibodies (mIgG, TRIM25) and lysates obtained from cells either expressing vtRNA1-1 (+) or not (-). Co-immunoprecipitation was assessed by qRT-PCR using primers specific for vtRNA1-1 or SNORD50A. Error bars indicate the standard deviation. ****p < 0.0001 (two-way ANOVA test) (G) Same as in (E) but using the siRNA targeting TRIM25.
(TIF)

S2 Fig (A) Northern blot analysis of the vtRNA1-1 expressed in the WT or vtRNA1-1 KO Huh7 cells after TRIM21 knockdown using the indicated probes. NC denotes the negative control (mock transfection). 5.8S rRNA served as a loading control. (B and C) Northern blot analysis using the indicated probes. After transfecting Huh7 cells with the siRNAs either targeting TRIM21 or TRIM25, total RNA was extracted and analyzed. (D) Northern blot analysis using the indicated probes (top) and immunoblot analysis using the indicated antibodies (middle). After transfecting Huh7 cells with the siRNA targeting TRIM21, cytoplasm/nucleus fractionation was performed. RNA and lysate were obtained from each fraction and analyzed. GAPDH served as a loading control. The intensity of vtRNA1-1 was normalized to that of 5.8S rRNA (bottom, n=3). Error bars indicate the standard deviation. *p < 0.05, **p < 0.01 (two-way ANOVA test) (E) Same as in (B) but using the siRNA targeting different sequence of TRIM21 mRNA. 5S rRNA served as a loading control. The intensity of vtRNA1-1 was normalized to that of 5S rRNA (right, n=3). Error bars indicate the standard deviation. *p < 0.05 (unpaired *t* test) (F) Northern blot analysis using the indicated probes (top) and immunoblot analysis using the indicated antibodies (bottom). After transfecting the indicated cells with the siRNA targeting TRIM21, total RNA and lysate were obtained and analyzed. (G) qPCR analysis of TRIM21 mRNA and vtRNA1-1 (n=4) after transfecting SNU423 or HepG2 cells with the siRNA targeting TRIM21. Error bars indicate the standard deviation. ****p < 0.0001 (two-way ANOVA test) (H) Same as in (F) but using the cervical cancer cell line HeLa. The intensity of vtRNA1-1 was normalized to that of 5.8S rRNA (right, n=3). Error bars indicate the standard deviation. ns: non-significant (unpaired *t* test). (I-K) Northern blot analysis using the indicated probes (top) and immunoblot analysis using the indicated antibodies (middle). After transfecting the indicated cells with a plasmid containing TRIM21 gene, total RNA and lysate were obtained and analyzed. EV denotes the empty vector (mock transfection). The intensity of vtRNA1-1 was normalized to that of 5.8S rRNA or 5S rRNA (n=4 for H, n=2 for I, and n=3 for J). Error bars indicate the standard deviation. ns: non-significant (unpaired *t* test).
(TIF)

S3 Fig (A) Northern blot analysis using the indicated probes (left, top), and immunoblot analysis using the indicated antibodies (left, bottom). After transfecting Huh7 cells with the siRNA targeting TRIM25, total RNA and lysate were obtained and analyzed. NC denotes the negative control (mock transfection). 5S rRNA and GAPDH served as RNA and protein loading controls, respectively. The intensity of indicated RNAs was normalized to that of 5S rRNA (right, n=4). Error bars indicate the standard deviation. ****p < 0.0001, ns: non-significant (two-way ANOVA test) (B) Northern blot analysis of the vtRNA1-1 expressed in the Huh7 cells after TRIM25 knockdown using the indicated probes. (C) Northern blot analysis of vtRNA1-1 and immunoblot analysis using the indicated antibodies (bottom). After transfecting the indicated cells with the siRNAs either targeting TRIM21 or TRIM25, total RNA and lysate were obtained and analyzed. The EtBr-stained gel (top) served as a loading control. (D) Northern blot analysis using the indicated probes (top) and immunoblot analysis using the indicated antibodies (bottom). After overexpressing TRIM25, total RNA and lysate were obtained and analyzed. EV denotes the empty vector (mock transfection). (E and F) Northern blot analysis using the indicated probes. After transfecting Huh7 cells with the siRNAs either targeting TRIM21, TRIM25, or both simultaneously (21&25) (E) or targeting TRIM2, TRIM26, or TRIM65 (F), total RNA was extracted and analyzed.
(TIF)

S4 Fig (A) Immunoblot analysis using the indicated antibodies to confirm the lysate with knocking down TRIM21 or TRIM25 for the *in vitro* RNA stability assay. After transfecting vtRNA1-1 KO Huh7 cells with the siRNAs either targeting TRIM21 or TRIM25, lysate was obtained and analyzed. GAPDH served as a loading control. NC denotes the negative control (mock transfection). (B) RNA stability assay followed by northern blot analysis using the indicated probes. After transfecting Huh7 cells with the siRNAs either targeting TRIM21 or TRIM25 and treating with RNA polymerase III inhibitor in the indicated times, total RNA was extracted and analyzed. 5.8S rRNA served as a loading control. (C) *In vitro* RNA stability assay followed by northern blot analysis using the indicated probes. Total RNA was extracted from Huh7 cells, mixed with lysate obtained from vtRNA1-1 KO Huh7 cells transfected with the siRNA targeting TRIM25, and incubated for the indicated time periods. RNA extracted from the mixtures was analyzed. (D) Same as in (C) but using total RNA extracted from vtRNA1-1 KO Huh7 cells expressing vtRNA1-1 exogenously.
(TIF)

S5 Fig (A) Sequences of vtRNA1-1 wild-type and the indicated mutants. (B-E) Northern blot analysis of the vtRNA1-1 mutants expressed in the vtRNA1-1 KO Huh7 cells after TRIM21 knockdown using the indicated probes. NC denotes the negative control (mock transfection). 5.8S rRNA served as a loading control. (F) Same as in (B) but using the siRNAs either targeting TRIM21 or TRIM25. The intensity of vtRNA1-1 mutants was normalized to that of 5.8S rRNA (right, n=3). Error bars indicate the standard deviation. **p < 0.01, ns: non-significant (two-way ANOVA test) (G-J) Same as in (B) but using the siRNAs either targeting TRIM21 or TRIM25. The EtBr-stained gel (bottom) served as a loading control. (K) Half-life of vtRNA1-1 M10 mutant calculated from qPCR analysis of cDNA synthesized from the same set of total RNA as in Fig 4D (n=3). ns: non-significant (unpaired *t* test) (L) *In vitro* RNA stability assay followed by northern blot analysis using the indicated probes. Total RNA was extracted from Huh7 cells expressing the vtRNA1-1 M10 mutant, mixed with lysate obtained from vtRNA1-1 KO Huh7 cells transfected with the siRNAs either targeting TRIM21 or TRIM25, and incubated for 0- or 30-min. RNA extracted from the mixtures was analyzed. (M) EMSA of radiolabeled vtRNA1-1 M10 mutant with increasing concentration of cell lysate obtained from control or TRIM21 siRNA-transfected vtRNA1-1 KO Huh7 cells.
(TIF)

S6 Fig (A) Immunoblot analysis using the indicated antibodies to confirm the knockdown for Fig 5A. After transfecting Huh7 cells with the siRNAs either targeting TRIM21, NSUN2, or both simultaneously (21&2), lysates were extracted and analyzed. NC denotes the negative control (mock transfection). GAPDH served as a loading control. (B) Northern blot analysis using the indicated probes. After transfecting vtRNA1-1 KO Huh7 cells with the siRNAs either targeting TRIM21, NSUN2, or both simultaneously (21&2) and plasmid containing vtRNA1-1 WT gene, total RNA was extracted and analyzed. 5.8S rRNA served as a loading control. (C) Sequences of vtRNA1-1 wild-type, C69A, and C69G. (D) Northern blot analysis using the indicated probes. After transfecting vtRNA1-1 KO Huh7 cells with plasmids containing vtRNA1-1 WT or the indicated mutant genes, total RNA was extracted and analyzed. (E) Northern blot analysis using the indicated probes. After transfecting vtRNA1-1 KO Huh7 cells with the siRNAs either targeting TRIM21 or TRIM25 and with a plasmid containing vtRNA1-1 WT or C69A mutant gene, total RNA was extracted and analyzed. (F) *In vitro* RNA stability assay followed by northern blot analysis using the indicated probes. Total RNA was extracted from Huh7 cells expressing the vtRNA1-1 C69A mutant, mixed with lysate obtained from vtRNA1-1 KO Huh7 cells transfected with the siRNA targeting TRIM21, and incubated for 0- or 30 min. RNA extracted from the mixtures was analyzed. (G) Northern blot analysis using the indicated probes (left). After transfecting vtRNA1-1 KO Huh7 cells with the siRNAs either targeting TRIM21 or TRIM25 and with the *in vitro*-transcribed (*iv*T) vtRNA1-1, total RNA was extracted and analyzed.
(TIF)

S7 Fig (A) Immunoblot analysis using the indicated antibodies (left). After transfecting SNU423 cells with the siRNA targeting TRIM21, lysates were obtained and analyzed. NC denotes the negative control (mock transfection). The intensity of indicated proteins was normalized to that of GAPDH (right, n=3). Error bars indicate the standard deviation. *p < 0.05, **p < 0.01 (two-way ANOVA test) (B) Same as in (A) but using Huh7 cells transfecting with the siRNA targeting TRIM25. The intensity of TRIM21 was normalized to that of GAPDH (right, n=4). Error bars indicate the standard deviation. ns: non-significant (unpaired *t* test) (C) Same as in (A) but using the siRNA targeting TRIM25. (D) Same as in (A) but using Huh7 cells transfecting with the siRNA targeting different sequence of TRIM21 mRNA. (E) qPCR analysis of RBCK1 mRNA and vtRNA1-1 (n=4) after transfecting Huh7 cells with the siRNA targeting RBCK1. Error bars indicate the standard deviation. ****p < 0.0001, ns: non-significant (two-way ANOVA test) (F) Northern blot analysis using the indicated probes (left, top), and immunoblot analysis using the indicated antibodies (left, bottom). After transfecting Huh7 cells with the siRNA targeting TRIM21 and treating with proteasomal inhibitor (Btz, bortezomib) or not, total RNA and lysate were obtained and analyzed. The intensity of vtRNA1-1 was normalized to that of 5.8S rRNA (right, n=4). Error bars indicate the standard deviation. *p < 0.05, **p < 0.01, ***p < 0.001, ns: non-significant (one-way ANOVA test) (G) Same as in (F) but using the siRNA targeting TRIM25.
(TIF)

S8 Fig (A) Northern blot analysis using the indicated probes. Total RNA was extracted from WT and vtRNA1-1 overexpressed Huh7 cells and analyzed. 5.8S rRNA served as a loading control. (B) Relative proliferation rates of WT and vtRNA1-1 overexpressed Huh7 cells were measured by the MTT assay (n=6). Error bars indicate the standard deviation. ***p < 0.001 (unpaired *t* test) (C) Relative cell number of WT and vtRNA1-1 overexpressed Huh7 cells was measured by cell counter (n=3). Error bars indicate the standard deviation. *p < 0.05, ns: non-significant (two-way ANOVA test) (D) Survival analysis of liver hepatocellular carcinoma patients based on the expression status of indicated single gene. Dataset was presented by TCGA database and analyzed using GEPIA analysis program (HR: hazards ratio). (E) Relative proliferation rates of SNU423 (left) and HepG2 (right) cells after transfecting with the siRNAs either targeting TRIM21 or TRIM25 were measured by the MTT assay (n=4). NC denotes the negative control (mock transfection). Error bars indicate the standard deviation. **p < 0.01, ***p < 0.001, ns: non-significant (unpaired *t* test) (F) Relative cell viabilities of Huh7 cells after treating sorafenib (24 hours, indicated concentrations) were measured by MTT assay (n=4). Error bars indicate the standard deviation. ****p < 0.0001 (one-way ANOVA test) (G and H) Relative cell viabilities of SNU423 (left) and HepG2 (right) cells after transfecting with the siRNAs either targeting TRIM21 or TRIM25 in the presence of sorafenib (24 hours, 12uM) were measured by MTT assay (n=5). Values were normalized to that of untreated cells. Error bars indicate the standard deviation. **p < 0.01, ****p < 0.0001, ns: non-significant (one-way ANOVA test) (I and J) Relative cell viabilities of vtRNA1-1 KO Huh7 cells transiently expressing vtRNA1-1 WT (I) or M10 (J) after transfecting with the siRNA TRIM21 in the presence of sorafenib (24 hours, 10 uM) or not (-) were measured by the MTT assay (n=3). Error bars indicate the standard deviation. **p < 0.01, ***p < 0.001, ns: non-significant (one-way ANOVA test).
(TIF)

S1 Table Probe sequences for northern blot. (DOCX)

S2 Table Primer sequences for RT-qPCR. (DOCX)

S3 Table Numerical values supporting each graph. (XLSX)
