# Supplementary figures and images for: TRIM21 modulates stability of pro-survival non-coding RNA vtRNA1–1 in human hepatocellular carcinoma cells
Source: PLoS Genet. 2025 Mar 17;21(3):e1011614. doi: 10.1371/journal.pgen.1011614 (PMC11940608; doi:10.1371/journal.pgen.1011614)

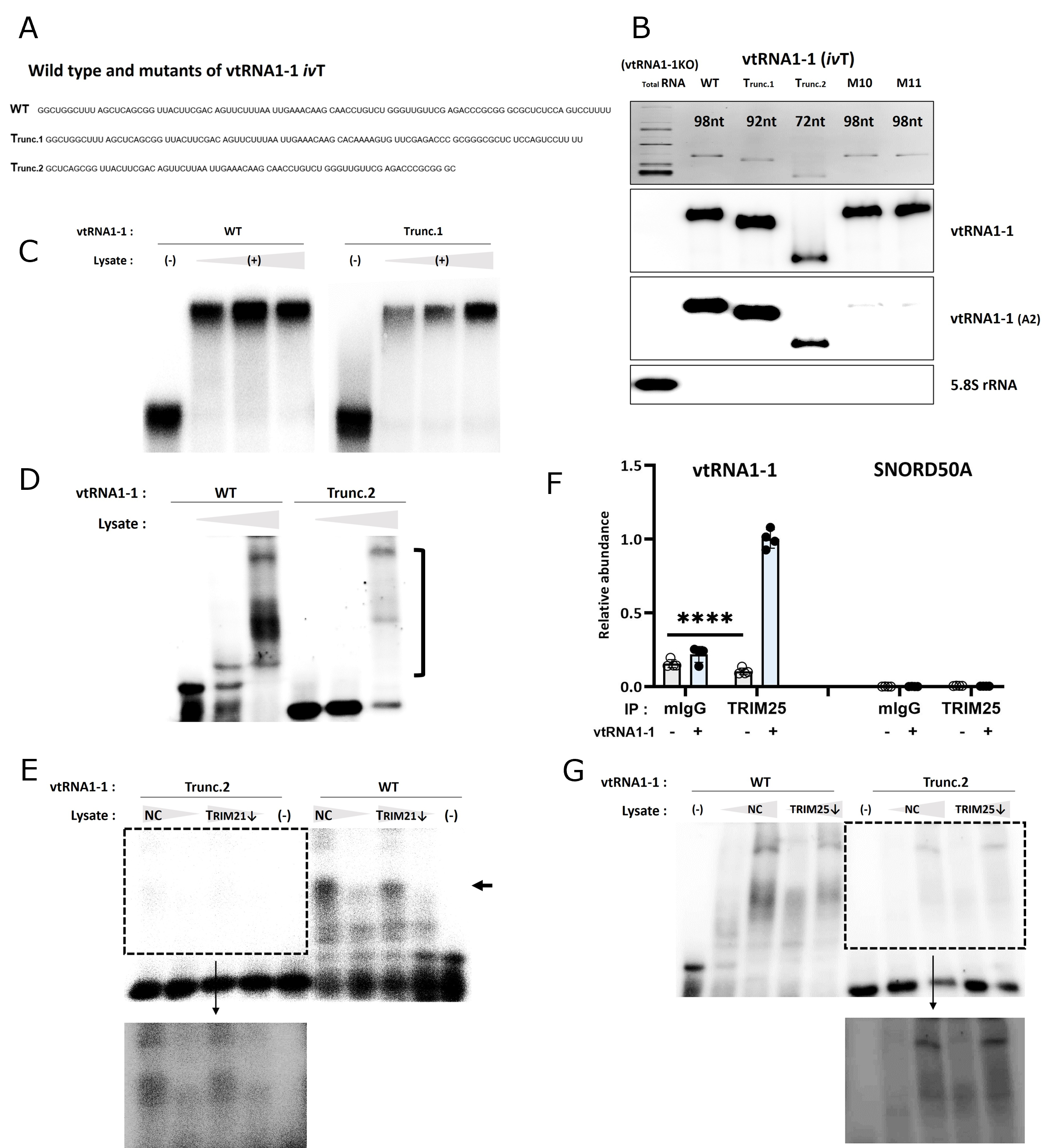

Supplement: S1 Fig — (A) Sequences of in vitro-transcribed vtRNA1-1 WT and truncated mutants (Trunc.1 and Trunc.2). (B) EtBr-stained gel and northern blot analysis using the indicated probes detecting in vitro-transcribed vtRNA1-1 WT and mutants. (C and D) EMSA of radiolabeled vtRNA1-1 WT or truncated mutants with increasing concentration of cell lysate obtained from vtRNA1-1 KO Huh7 cells. (E) EMSA of radiolabeled vtRNA1-1 WT or truncated mutant (Trunc.2) with increasing concentration of cell lysate obtained from control or TRIM21 siRNAs-transfected vtRNA1-1 KO Huh7 cells (top). The arrow marks the upshifted RNP. (F) fCLIP using the indicated antibodies (mIgG, TRIM25) and lysates obtained from cells either expressing vtRNA1-1 (+) or not (-). Co-immunoprecipitation was assessed by qRT-PCR using primers specific for vtRNA1-1 or SNORD50A. Error bars indicate the standard deviation. ****p < 0.0001 (two-way ANOVA test) (G) Same as in (E) but using the siRNA targeting TRIM25. (TIF) [file pgen.1011614.s001.tif]

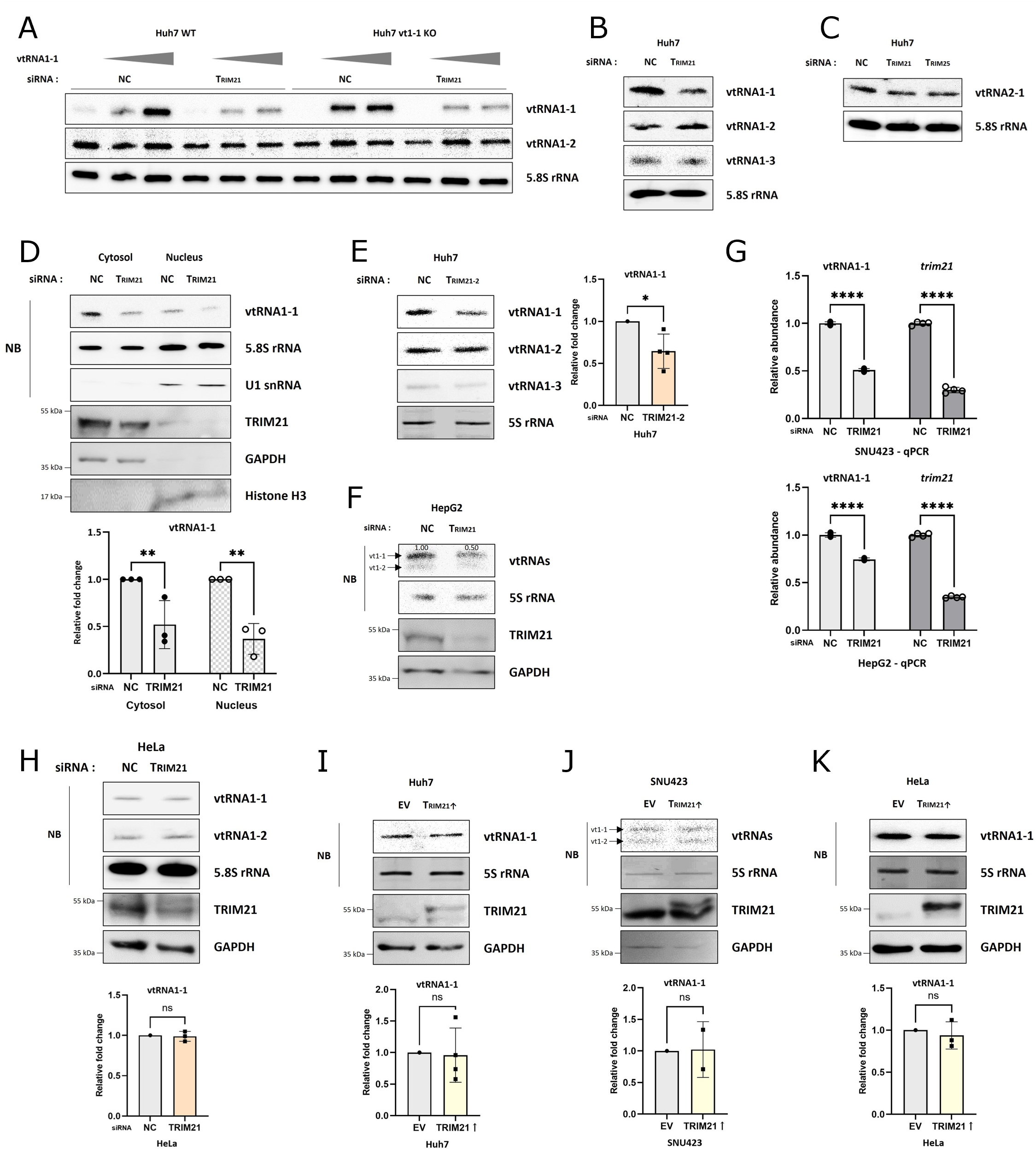

Supplement: S2 Fig — (A) Northern blot analysis of the vtRNA1-1 expressed in the WT or vtRNA1-1 KO Huh7 cells after TRIM21 knockdown using the indicated probes. NC denotes the negative control (mock transfection). 5.8S rRNA served as a loading control. (B and C) Northern blot analysis using the indicated probes. After transfecting Huh7 cells with the siRNAs either targeting TRIM21 or TRIM25, total RNA was extracted and analyzed. (D) Northern blot analysis using the indicated probes (top) and immunoblot analysis using the indicated antibodies (middle). After transfecting Huh7 cells with the siRNA targeting TRIM21, cytoplasm/nucleus fractionation was performed. RNA and lysate were obtained from each fraction and analyzed. GAPDH served as a loading control. The intensity of vtRNA1-1 was normalized to that of 5.8S rRNA (bottom, n=3). Error bars indicate the standard deviation. *p < 0.05, **p < 0.01 (two-way ANOVA test) (E) Same as in (B) but using the siRNA targeting different sequence of TRIM21 mRNA. 5S rRNA served as a loading control. The intensity of vtRNA1-1 was normalized to that of 5S rRNA (right, n=3). Error bars indicate the standard deviation. *p < 0.05 (unpaired t test) (F) Northern blot analysis using the indicated probes (top) and immunoblot analysis using the indicated antibodies (bottom). After transfecting the indicated cells with the siRNA targeting TRIM21, total RNA and lysate were obtained and analyzed. (G) qPCR analysis of TRIM21 mRNA and vtRNA1-1 (n=4) after transfecting SNU423 or HepG2 cells with the siRNA targeting TRIM21. Error bars indicate the standard deviation. ****p < 0.0001 (two-way ANOVA test) (H) Same as in (F) but using the cervical cancer cell line HeLa. The intensity of vtRNA1-1 was normalized to that of 5.8S rRNA (right, n=3). Error bars indicate the standard deviation. ns: non-significant (unpaired t test). (I-K) Northern blot analysis using the indicated probes (top) and immunoblot analysis using the indicated antibodies (middle). After transfecting the [file pgen.1011614.s002.tif]

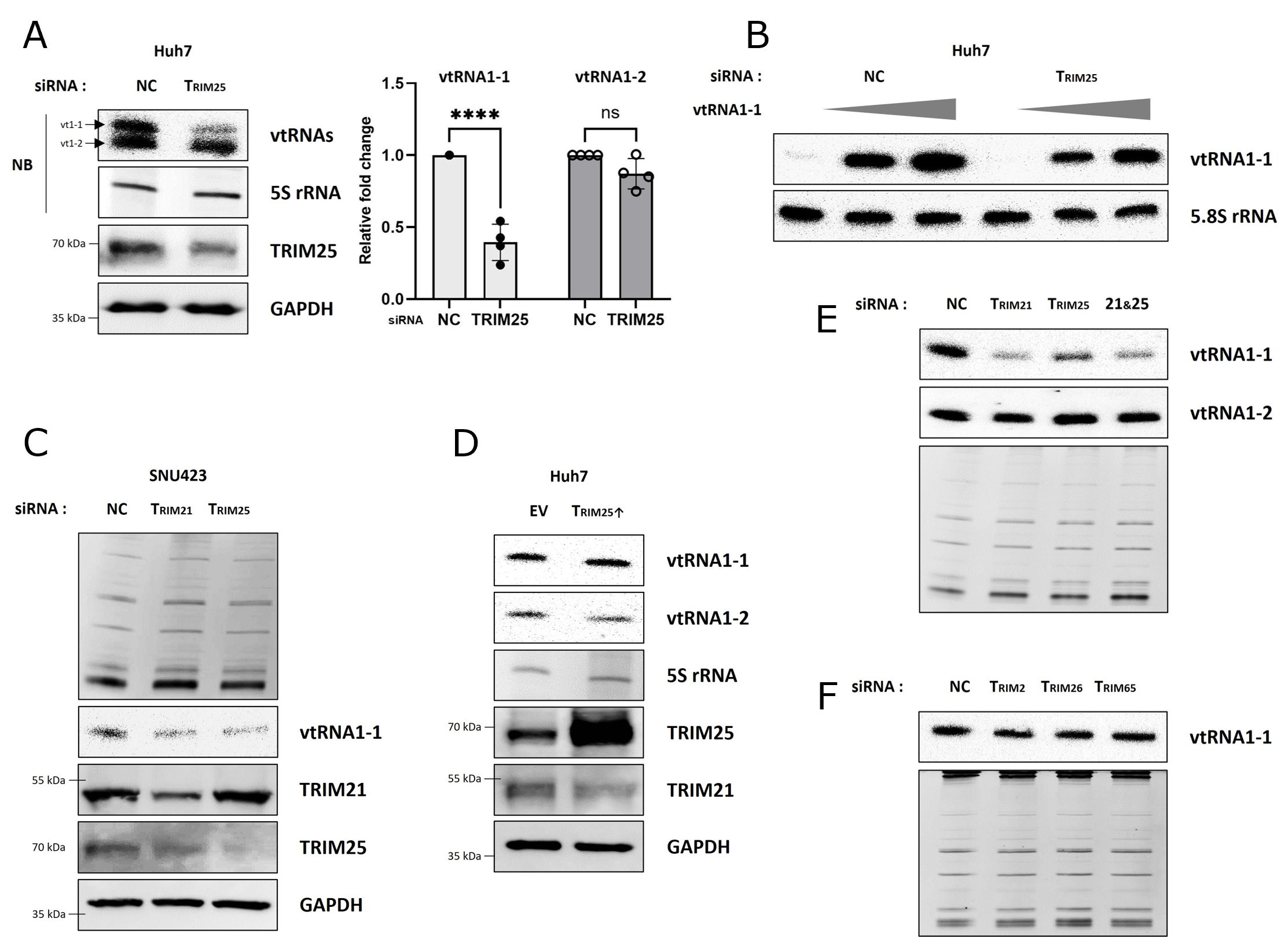

Supplement: S3 Fig — (A) Northern blot analysis using the indicated probes (left, top), and immunoblot analysis using the indicated antibodies (left, bottom). After transfecting Huh7 cells with the siRNA targeting TRIM25, total RNA and lysate were obtained and analyzed. NC denotes the negative control (mock transfection). 5S rRNA and GAPDH served as RNA and protein loading controls, respectively. The intensity of indicated RNAs was normalized to that of 5S rRNA (right, n=4). Error bars indicate the standard deviation. ****p < 0.0001, ns: non-significant (two-way ANOVA test) (B) Northern blot analysis of the vtRNA1-1 expressed in the Huh7 cells after TRIM25 knockdown using the indicated probes. (C) Northern blot analysis of vtRNA1-1 and immunoblot analysis using the indicated antibodies (bottom). After transfecting the indicated cells with the siRNAs either targeting TRIM21 or TRIM25, total RNA and lysate were obtained and analyzed. The EtBr-stained gel (top) served as a loading control. (D) Northern blot analysis using the indicated probes (top) and immunoblot analysis using the indicated antibodies (bottom). After overexpressing TRIM25, total RNA and lysate were obtained and analyzed. EV denotes the empty vector (mock transfection). (E and F) Northern blot analysis using the indicated probes. After transfecting Huh7 cells with the siRNAs either targeting TRIM21, TRIM25, or both simultaneously (21&25) (E) or targeting TRIM2, TRIM26, or TRIM65 (F), total RNA was extracted and analyzed. (TIF) [file pgen.1011614.s003.tif]

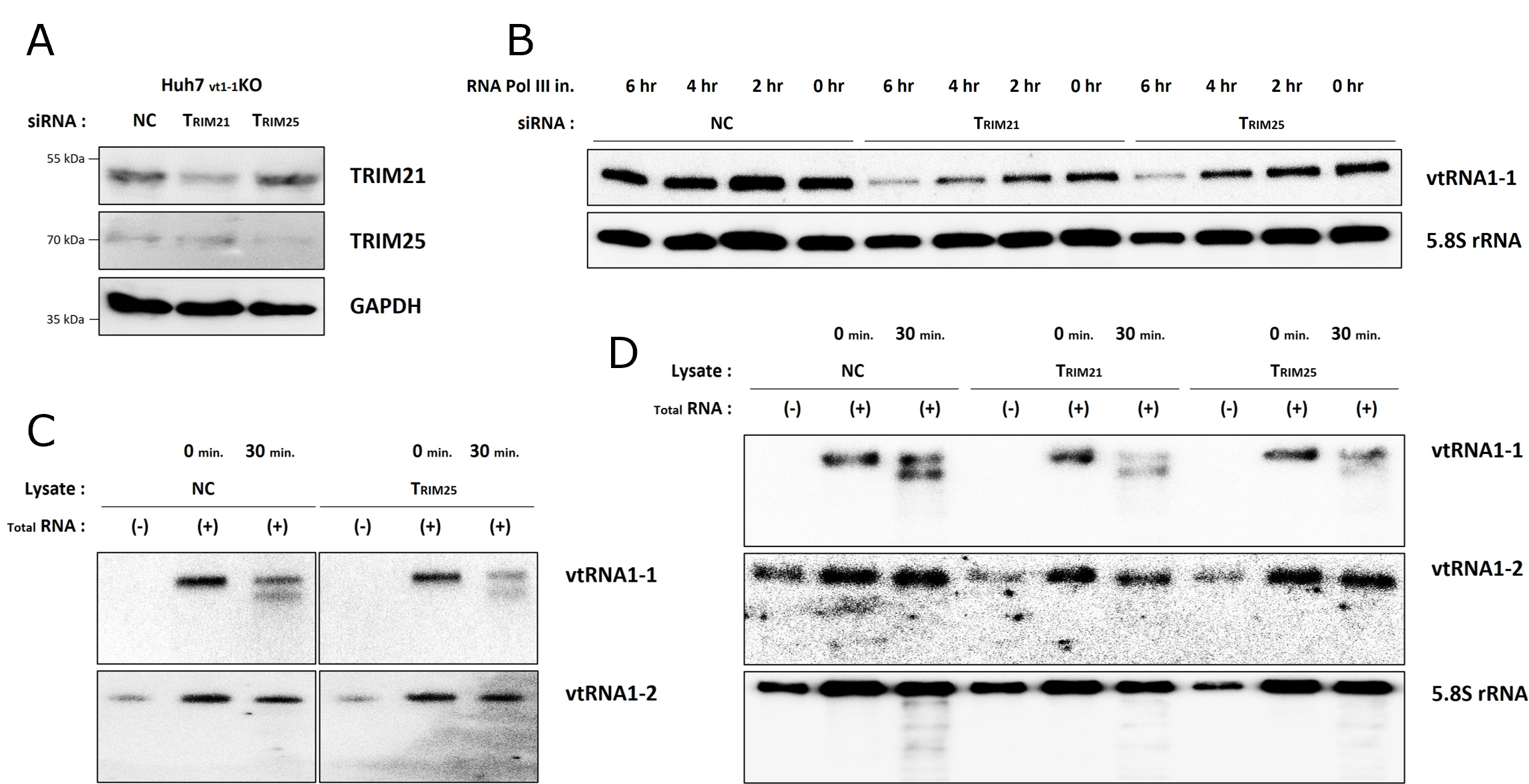

Supplement: S4 Fig — (A) Immunoblot analysis using the indicated antibodies to confirm the lysate with knocking down TRIM21 or TRIM25 for the in vitro RNA stability assay. After transfecting vtRNA1-1 KO Huh7 cells with the siRNAs either targeting TRIM21 or TRIM25, lysate was obtained and analyzed. GAPDH served as a loading control. NC denotes the negative control (mock transfection). (B) RNA stability assay followed by northern blot analysis using the indicated probes. After transfecting Huh7 cells with the siRNAs either targeting TRIM21 or TRIM25 and treating with RNA polymerase III inhibitor in the indicated times, total RNA was extracted and analyzed. 5.8S rRNA served as a loading control. (C) In vitro RNA stability assay followed by northern blot analysis using the indicated probes. Total RNA was extracted from Huh7 cells, mixed with lysate obtained from vtRNA1-1 KO Huh7 cells transfected with the siRNA targeting TRIM25, and incubated for the indicated time periods. RNA extracted from the mixtures was analyzed. (D) Same as in (C) but using total RNA extracted from vtRNA1-1 KO Huh7 cells expressing vtRNA1-1 exogenously. (TIF) [file pgen.1011614.s004.tif]

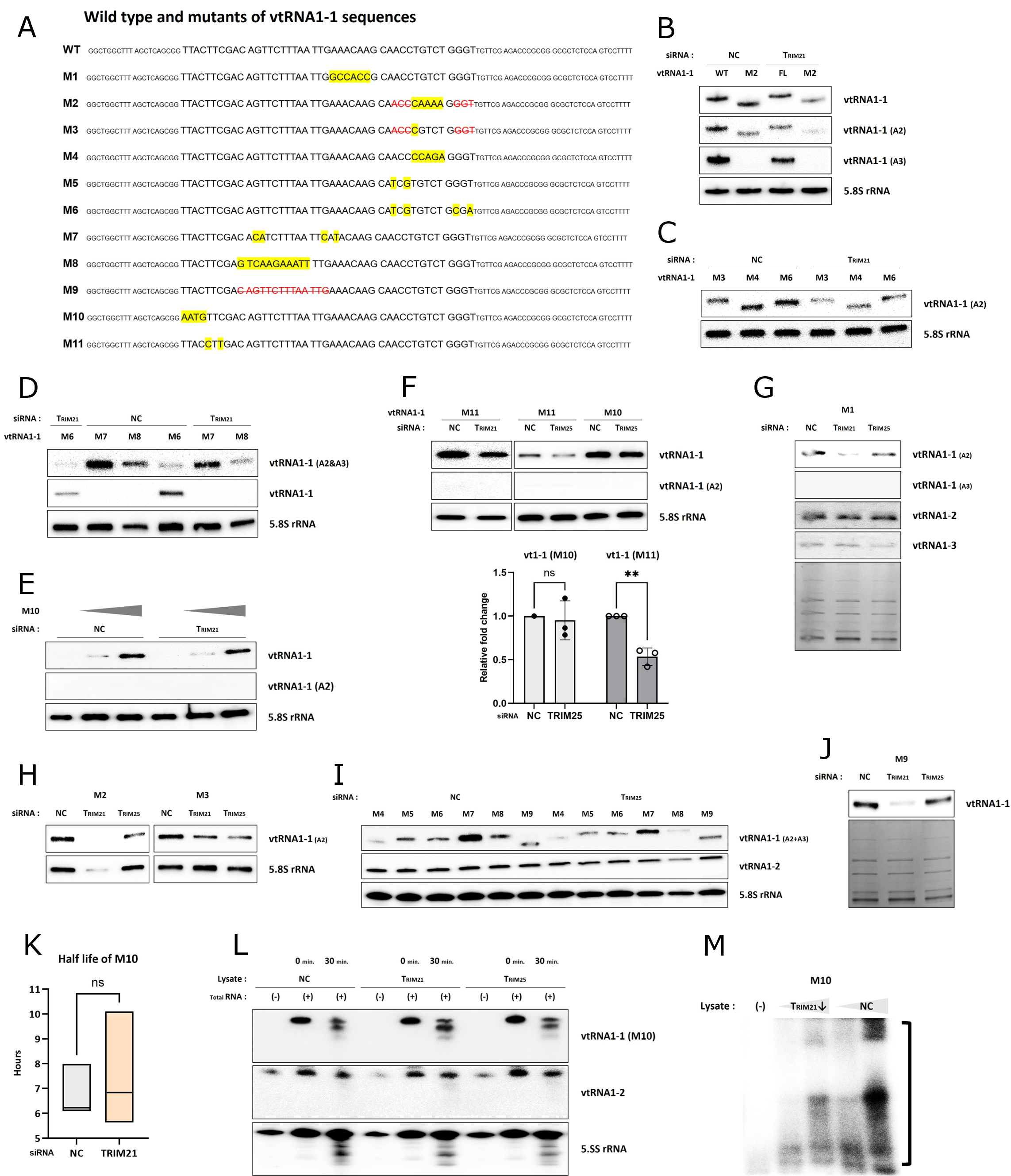

Supplement: S5 Fig — (A) Sequences of vtRNA1-1 wild-type and the indicated mutants. (B-E) Northern blot analysis of the vtRNA1-1 mutants expressed in the vtRNA1-1 KO Huh7 cells after TRIM21 knockdown using the indicated probes. NC denotes the negative control (mock transfection). 5.8S rRNA served as a loading control. (F) Same as in (B) but using the siRNAs either targeting TRIM21 or TRIM25. The intensity of vtRNA1-1 mutants was normalized to that of 5.8S rRNA (right, n=3). Error bars indicate the standard deviation. **p < 0.01, ns: non-significant (two-way ANOVA test) (G-J) Same as in (B) but using the siRNAs either targeting TRIM21 or TRIM25. The EtBr-stained gel (bottom) served as a loading control. (K) Half-life of vtRNA1-1 M10 mutant calculated from qPCR analysis of cDNA synthesized from the same set of total RNA as in Fig 4D (n=3). ns: non-significant (unpaired t test) (L) In vitro RNA stability assay followed by northern blot analysis using the indicated probes. Total RNA was extracted from Huh7 cells expressing the vtRNA1-1 M10 mutant, mixed with lysate obtained from vtRNA1-1 KO Huh7 cells transfected with the siRNAs either targeting TRIM21 or TRIM25, and incubated for 0- or 30-min. RNA extracted from the mixtures was analyzed. (M) EMSA of radiolabeled vtRNA1-1 M10 mutant with increasing concentration of cell lysate obtained from control or TRIM21 siRNA-transfected vtRNA1-1 KO Huh7 cells. (TIF) [file pgen.1011614.s005.tif]

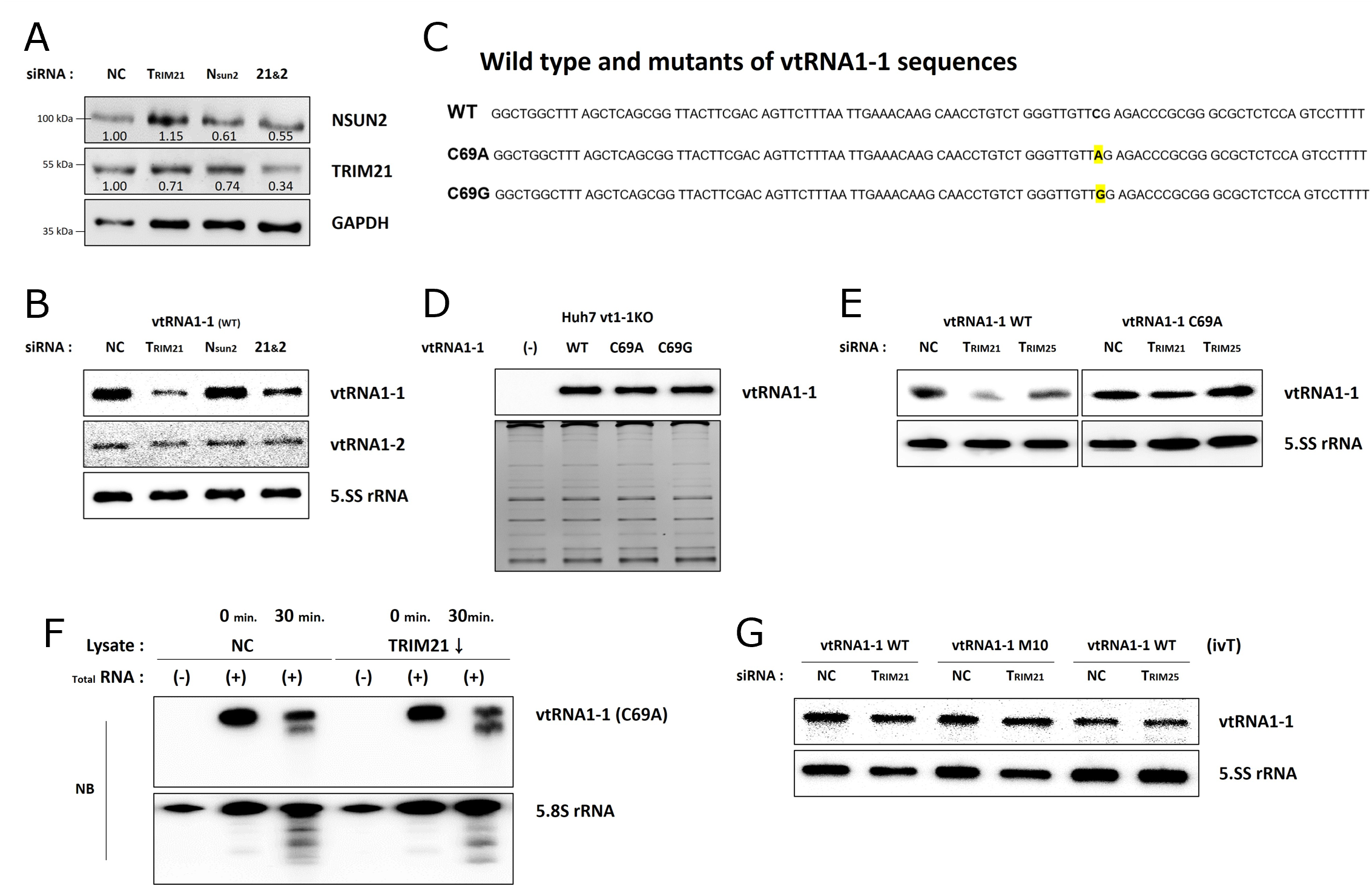

Supplement: S6 Fig — (A) Immunoblot analysis using the indicated antibodies to confirm the knockdown for Fig 5A. After transfecting Huh7 cells with the siRNAs either targeting TRIM21, NSUN2, or both simultaneously (21&2), lysates were extracted and analyzed. NC denotes the negative control (mock transfection). GAPDH served as a loading control. (B) Northern blot analysis using the indicated probes. After transfecting vtRNA1-1 KO Huh7 cells with the siRNAs either targeting TRIM21, NSUN2, or both simultaneously (21&2) and plasmid containing vtRNA1-1 WT gene, total RNA was extracted and analyzed. 5.8S rRNA served as a loading control. (C) Sequences of vtRNA1-1 wild-type, C69A, and C69G. (D) Northern blot analysis using the indicated probes. After transfecting vtRNA1-1 KO Huh7 cells with plasmids containing vtRNA1-1 WT or the indicated mutant genes, total RNA was extracted and analyzed. (E) Northern blot analysis using the indicated probes. After transfecting vtRNA1-1 KO Huh7 cells with the siRNAs either targeting TRIM21 or TRIM25 and with a plasmid containing vtRNA1-1 WT or C69A mutant gene, total RNA was extracted and analyzed. (F) In vitro RNA stability assay followed by northern blot analysis using the indicated probes. Total RNA was extracted from Huh7 cells expressing the vtRNA1-1 C69A mutant, mixed with lysate obtained from vtRNA1-1 KO Huh7 cells transfected with the siRNA targeting TRIM21, and incubated for 0- or 30 min. RNA extracted from the mixtures was analyzed. (G) Northern blot analysis using the indicated probes (left). After transfecting vtRNA1-1 KO Huh7 cells with the siRNAs either targeting TRIM21 or TRIM25 and with the in vitro-transcribed (ivT) vtRNA1-1, total RNA was extracted and analyzed. (TIF) [file pgen.1011614.s006.tif]

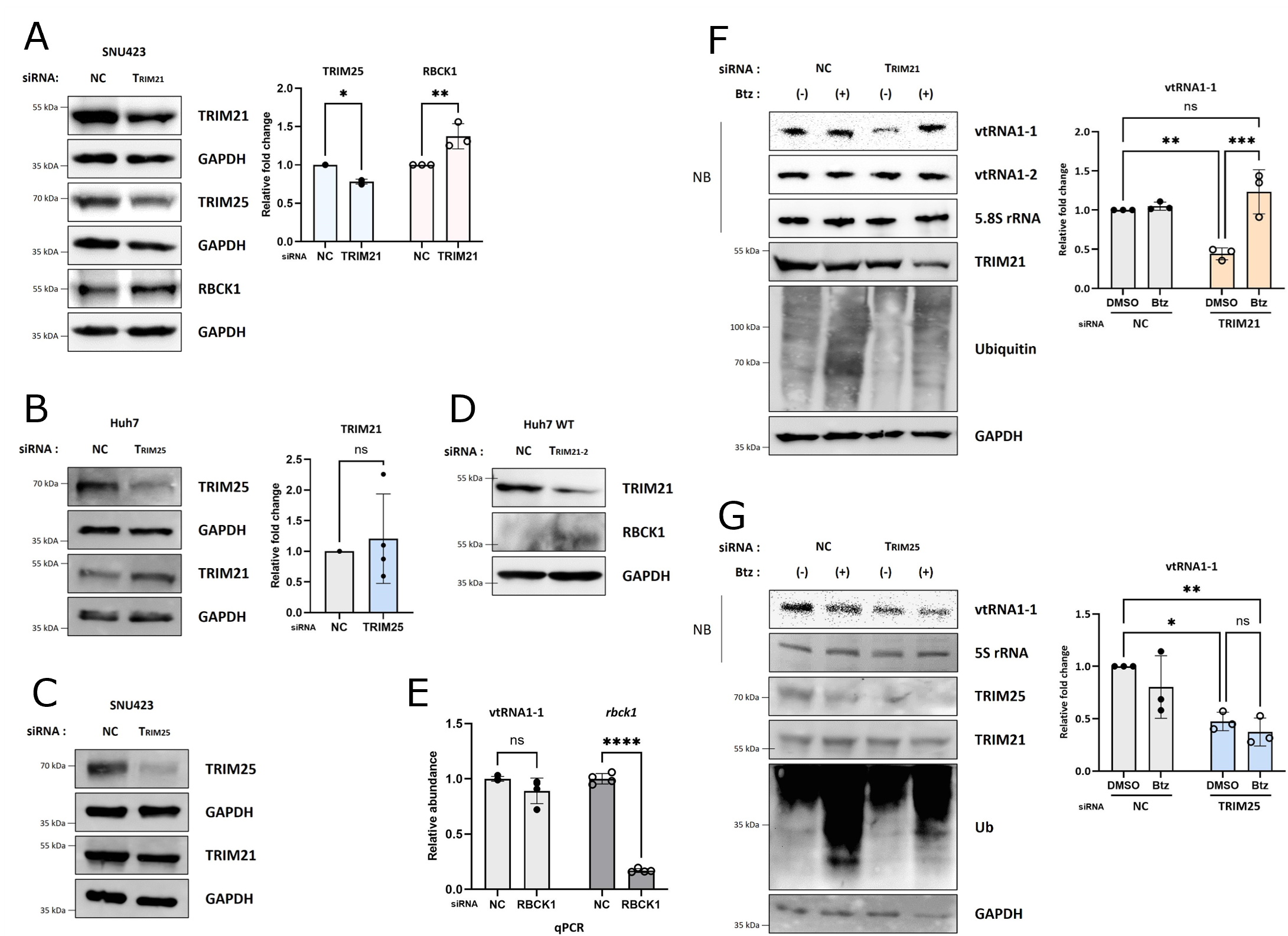

Supplement: S7 Fig — (A) Immunoblot analysis using the indicated antibodies (left). After transfecting SNU423 cells with the siRNA targeting TRIM21, lysates were obtained and analyzed. NC denotes the negative control (mock transfection). The intensity of indicated proteins was normalized to that of GAPDH (right, n=3). Error bars indicate the standard deviation. *p < 0.05, **p < 0.01 (two-way ANOVA test) (B) Same as in (A) but using Huh7 cells transfecting with the siRNA targeting TRIM25. The intensity of TRIM21 was normalized to that of GAPDH (right, n=4). Error bars indicate the standard deviation. ns: non-significant (unpaired t test) (C) Same as in (A) but using the siRNA targeting TRIM25. (D) Same as in (A) but using Huh7 cells transfecting with the siRNA targeting different sequence of TRIM21 mRNA. (E) qPCR analysis of RBCK1 mRNA and vtRNA1-1 (n=4) after transfecting Huh7 cells with the siRNA targeting RBCK1. Error bars indicate the standard deviation. ****p < 0.0001, ns: non-significant (two-way ANOVA test) (F) Northern blot analysis using the indicated probes (left, top), and immunoblot analysis using the indicated antibodies (left, bottom). After transfecting Huh7 cells with the siRNA targeting TRIM21 and treating with proteasomal inhibitor (Btz, bortezomib) or not, total RNA and lysate were obtained and analyzed. The intensity of vtRNA1-1 was normalized to that of 5.8S rRNA (right, n=4). Error bars indicate the standard deviation. *p < 0.05, **p < 0.01, ***p < 0.001, ns: non-significant (one-way ANOVA test) (G) Same as in (F) but using the siRNA targeting TRIM25. (TIF) [file pgen.1011614.s007.tif]

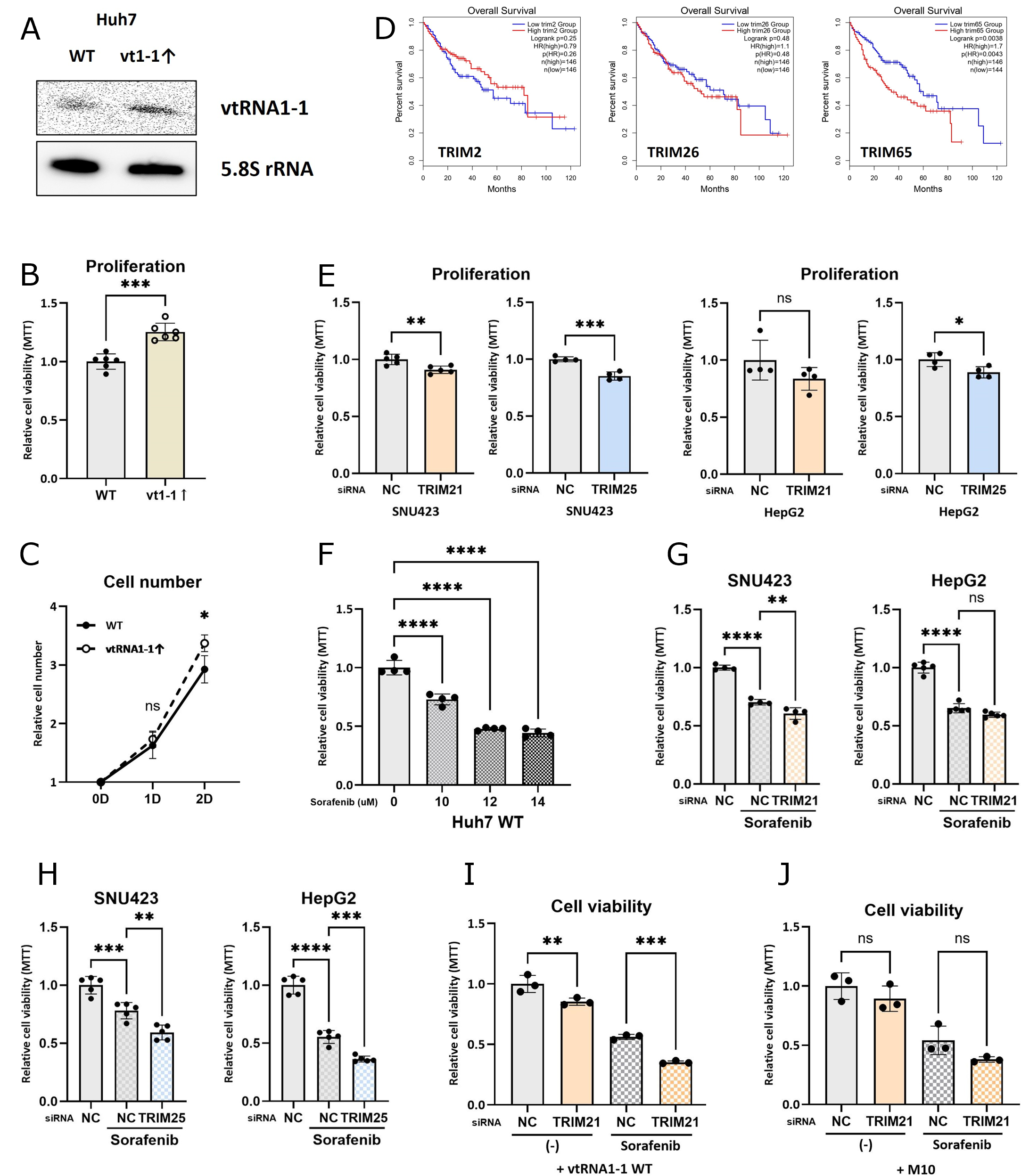

Supplement: S8 Fig — (A) Northern blot analysis using the indicated probes. Total RNA was extracted from WT and vtRNA1-1 overexpressed Huh7 cells and analyzed. 5.8S rRNA served as a loading control. (B) Relative proliferation rates of WT and vtRNA1-1 overexpressed Huh7 cells were measured by the MTT assay (n=6). Error bars indicate the standard deviation. ***p < 0.001 (unpaired t test) (C) Relative cell number of WT and vtRNA1-1 overexpressed Huh7 cells was measured by cell counter (n=3). Error bars indicate the standard deviation. *p < 0.05, ns: non-significant (two-way ANOVA test) (D) Survival analysis of liver hepatocellular carcinoma patients based on the expression status of indicated single gene. Dataset was presented by TCGA database and analyzed using GEPIA analysis program (HR: hazards ratio). (E) Relative proliferation rates of SNU423 (left) and HepG2 (right) cells after transfecting with the siRNAs either targeting TRIM21 or TRIM25 were measured by the MTT assay (n=4). NC denotes the negative control (mock transfection). Error bars indicate the standard deviation. **p < 0.01, ***p < 0.001, ns: non-significant (unpaired t test) (F) Relative cell viabilities of Huh7 cells after treating sorafenib (24 hours, indicated concentrations) were measured by MTT assay (n=4). Error bars indicate the standard deviation. ****p < 0.0001 (one-way ANOVA test) (G and H) Relative cell viabilities of SNU423 (left) and HepG2 (right) cells after transfecting with the siRNAs either targeting TRIM21 or TRIM25 in the presence of sorafenib (24 hours, 12uM) were measured by MTT assay (n=5). Values were normalized to that of untreated cells. Error bars indicate the standard deviation. **p < 0.01, ****p < 0.0001, ns: non-significant (one-way ANOVA test) (I and J) Relative cell viabilities of vtRNA1-1 KO Huh7 cells transiently expressing vtRNA1-1 WT (I) or M10 (J) after transfecting with the siRNA TRIM21 in the presence of sorafenib (24 hours, 10 uM) or not (-) were measured by the MTT assay (n=3). Error [file pgen.1011614.s008.tif]
