# Supplementary material for: TRIM21 modulates stability of pro-survival non-coding RNA vtRNA1–1 in human hepatocellular carcinoma cells
Source: PLoS Genet. 2025 Mar 17;21(3):e1011614. doi: 10.1371/journal.pgen.1011614 (PMC11940608; doi:10.1371/journal.pgen.1011614)
Supplement: S1 Table — (DOCX) [file pgen.1011614.s009.docx]

**S1_Table: Probe sequences for northern blot**

| Probe (NB) | Sequence (5' - 3') |
| --- | --- |
| vtRNA1-1 (NB) | GCTTGTTTCAATTAAAGAACTGTCG |
| vtRNA1-1 (A1) | AGTAACCGCTGAGCTAAAG |
| vtRNA1-1 (A2) | AAAGAACTGTCGAAGTAACC |
| vtRNA1-1 (A3) | CCAGACAGGTTGCTTGTT |
| vtRNA1-1 (A4) | CCCGCGGGTCTCGAACAA |
| vtRNA1-1 (3') | CGCGAGAGGTCAGGTTTT |
| vtRNA1-2 | AGGTGGTTACAATGTACTCGAAG |
| vtRNA1-3 | GAGGTGGTTTGATGACACGCGAA |
| vtRNA2-1 | TTGCATAAAAGGGTCAGTAAGCT |
| 5.8S rRNA | TCCTGCAATTCACATTAATTCTCGAGCTAGC |
| U1 snRNA | CAATGGATAAGCCTCGCCCT |
