# Supplementary material for: TRIM21 modulates stability of pro-survival non-coding RNA vtRNA1–1 in human hepatocellular carcinoma cells
Source: PLoS Genet. 2025 Mar 17;21(3):e1011614. doi: 10.1371/journal.pgen.1011614 (PMC11940608; doi:10.1371/journal.pgen.1011614)
Supplement: S2 Table — (DOCX) [file pgen.1011614.s010.docx]

**S2_Table: Primer sequences for RT-qPCR**

| Primer (qPCR) | Sequence (5' - 3') |
| --- | --- |
| vtRNA1 (Fw) | GGCTGGCTTTAGCTCAGC |
| vtRNA1-1 (Rv) | CCAGACAGGTTGCTTGTT |
| vtRNA1-2 (RV) | AGGTGGTTACAATGTACTCGAAG |
| vtRNA1-3 (RV) | GAGGTGGTTTGATGACACGCGAA |
| 18S rRNA (Fw) | GAGAAACGGCTACCACATCCA |
| 18S rRNA (RV) | CTCCAATGGATCCTCGTTAAAGG |
| TRIM21 (Fw) | CCTTCTTCAGTCCTGGTTTCAATGATG |
| TRIM21 (Rv) | TCAATAGTCAGTGGATCCTTGTGATCC |
| TRIM25 (Fw) | GACCACGGCTTTGTCATCTTCTT |
| TRIM25 (Rv) | AGTCCACCCTGAACTTATACATCAGG |
| SNORD50a (Fw) | TATCTGTGATGATCTTATCCCGAACC |
| SNORD50a (Rv) | ATCTCAGAAGCCAGATCCGT |
| SNORD10 (Fw) | GCTCTGTGATGGAGCCC |
| SNORD10 (Rv) | TAGTCTGCTCTCGAGGTACAAAGAC |
